# Study on the Structural Characteristics of Narrow Fractions of Catalytic Cracking Slurry and the Formation Pathway of Mesophase Pitch

**DOI:** 10.3390/ma19122528

**Published:** 2026-06-11

**Authors:** Xuesong Shan, Shuandi Hou, Renqing Chu, Yun Wu, Yuanyuan Zhang, Dan Guo, Yongen Gao, Shiwen Li, Zihui Ma

**Affiliations:** 1 SINOPEC Research Institute of Petroleum Processing Co., Ltd., Beijing 100083, China; shanxuesong.ripp@sinopec.com; 2 SINOPEC Dalian Petrochemical Research Institute Co., Ltd., Dalian 116045, China; churenqing.fshy@sinopec.com (R.C.); wuyun.fshy@sinopec.com (Y.W.); zhangyuanyuan.fshy@sinopec.com (Y.Z.); guodan.fshy@sinopec.com (D.G.); gaoyongen.fshy@sinopec.com (Y.G.); lishiwen.fshy@sinopec.com (S.L.); mazihui.fshy@sinopec.com (Z.M.)

**Keywords:** mesophase pitch, catalytic cracking slurry, narrow fraction, liquid-phase carbonization, formation mechanism

## Abstract

FDO’s wide boiling range and complex composition hinder controlled synthesis of high-performance mesophase pitch. Here, FDO was separated into light, middle, and heavy narrow fractions by vacuum distillation. Multi-scale characterization traced molecular evolution and mesophase development. The light fraction consists of three-ring aromatics with short alkyl side chains and shows the lowest reactivity, yielding limited condensation and poor stacking with isotropic regions and dispersed spheres. The middle fraction contains four-ring aromatics with moderately extended chains, exhibiting enhanced reactivity and undergoing nucleation, growth, coalescence, and disintegration of mesophase spheres. However, insufficient volatiles restrict shear orientation, forming a mosaic texture. The heavy fraction has four-ring aromatics with the longest alkyl chains and the lowest substitution degree, giving the highest reactivity. During thermal cracking, long chains release abundant radicals and volatiles; directional escape generates shear, promoting rapid growth and ordered alignment of aromatic lamellae. At 440 °C for 12 h, this fraction yields high-quality mesophase pitch with small-domain texture, a low softening point (295 °C), and high anisotropic content (98.8%). The pitch shows excellent spinnability, and derived carbon fibers (tensile strength ~1.45 GPa, modulus ~151 GPa) outperform a commercial reference processed under identical conditions. This study reveals molecular-level regulation of mesophase evolution by narrow fraction structures.

## 1. Introduction

The fluidized catalytic cracking decant oil (FCC decant oil, FDO) is a heavy byproduct generated during the fluidized catalytic cracking process. Its molecular structure contains a high proportion of 2–5 ring aromatics with low heteroatom content, enabling the formation of planar aromatic macromolecules rich in sp2 hybridized carbon. These planar molecules stack vertically through van der Waals forces [1], rendering FDO a promising and cost-effective precursor for the synthesis of carbonaceous materials. Mesophase pitch is a nematic liquid crystal precursor formed by the layered stacking of polycyclic aromatic hydrocarbons (PAHs). Due to its high carbon content, excellent graphitization ability, and tunable molecular orientation, it is widely used as a precursor for high-performance carbon materials such as high-performance carbon fibers [2,3,4], needle coke [5], foam carbon [6], and lithium-ion battery anode materials [7]. Converting low-value FDO into high-quality mesophase pitch represents a crucial pathway for the value-added utilization of heavy oil resources.

The properties of mesophase pitch–particularly its optical texture, spinnability, and subsequent graphitizability–are inherently governed by the molecular structure and chemical composition of the precursor, as well as by the liquid-phase carbonization conditions [8,9,10,11]. Ideally, the mesophase pitch precursor should contain planar polycyclic aromatic hydrocarbons with moderate reactivity to facilitate ordered molecular stacking and growth, thereby fostering the development of large anisotropic domains. FCC decant oil (FDO), which is rich in aliphatic polycyclic aromatic hydrocarbon structures, appears to meet this requirement. However, FDO is not only complex in composition, consisting of a mixture of thousands of compounds, but also has a wide boiling range, covering various aromatic hydrocarbons ranging from monocyclic to polycyclic, paraffins, naphthenes, and heteroatomic compounds containing sulfur, nitrogen, and oxygen. This high molecular heterogeneity leads to different thermal reaction activities of different components under the same carbonization conditions. Therefore, the formation and growth of mesophase pitch are not consistent in the kinetic pathway, often resulting in low anisotropic content in the product, heterogeneous optical texture, and poor spinnability [12], which severely restricts its application in high-performance carbon materials.

To address this issue, the separation of FDO into narrow fractions has been proposed as an effective pretreatment strategy to homogenize the molecular weight [12,13] and structural distribution [14,15] of the feedstock. Studies have shown that optimizing the molecular weight distribution of the feedstock by cutting its boiling range can promote uniform condensation of aromatic molecules, thereby avoiding the possibility of excessive condensation reactions that produce macromolecules with poor planarity [16]. Lin et al. [17] systematically investigated the effects of thermal treatment on catalytic cracking slurry and its five narrow fractions, revealing that mild thermal treatment improves the thermal stability of the middle boiling point fraction by converting low-reactivity components into heavy components. Subsequent carbonization experiments showed that narrow fractions with higher thermal stability can produce needle coke with superior optical texture and better-developed mesophase. Ge et al. [18] separated ethylene tar into light and heavy fractions through vacuum distillation and examined the influence of distillation temperature on the composition of the heavy fraction. They found that HC-250 obtained at a distillation temperature of 250 °C is rich in 3- to 5-ring aromatic hydrocarbons, exhibiting optimal thermal reactivity and mesophase development capability. Upon thermal treatment at 400 °C for 12 h under atmospheric pressure, this fraction yielded a high-performance mesophase pitch with 100% anisotropy, a high softening point (299 °C), and a high carbon yield (75.4%), demonstrating that regulating the composition of narrow fractions via distillation is an effective approach to the high-value utilization of ethylene tar. Li et al. [14] separated FDO into light, medium, and heavy fractions through vacuum distillation, and systematically investigated the influence of a boron trifluoride complex catalyst on the liquid-phase carbonization behavior of each fraction. Their results revealed that the heavy fraction, enriched in aromatics with four or more rings, exhibited superior catalytic activity and yielded a high-performance mesophase pitch with a mesophase content of up to 95.3%. However, the light fraction rich in 1~3 ring aromatic hydrocarbons formed a disordered cross-linked structure due to the introduction of oxygen-containing groups, resulting in almost complete isotropy of the product. These findings revealed the catalytic activity differences between different fraction compositions. Although these works have confirmed the effective role of fraction separation, their research focus has mainly been on optimizing the process parameters of specific fractions or comparing the properties of final products. Consequently, there remains a lack of systematic mechanistic analysis regarding how the distinct molecular structures of different narrow fractions regulate the mesophase evolution pathway, encompassing the complete sequence from nucleation, growth, and polycondensation to the eventual development of optical texture. This knowledge gap constrains the ability to achieve controlled synthesis of high-performance mesophase pitch based on the molecular characteristics of the feedstock.

Thus, the study systematically investigates the regulatory mechanism by which the molecular characteristics of individual narrow fractions derived from FDO govern the formation pathway of their resulting mesophase pitches. Through precise fractionation, FDO was separated into three narrow fractions with distinct boiling range gradients (light fraction, medium fraction, and heavy fraction). Under parallel and controllable thermal polycondensation conditions, a comprehensive comparative study was systematically conducted from raw materials to products. The molecular structures of the three narrow fractions and their derived mesophase pitch were characterized by multiple analytical techniques, including elemental analysis, FT-IR, 1H NMR, 13C NMR, MALDI-TOF MS, XRD, and Raman spectroscopy. Based on a systematic comparison of the liquid-phase carbonization behaviors of the three narrow fractions at the same temperature, the dynamic evolution pathway of their molecular structure of each narrow fraction during thermal conversion was further investigated. The correlation between the chemical structure of the narrow fraction raw materials and the structure and properties of mesophase pitch was explored at the molecular level, elucidating the decisive role of fraction differences in determining the final mesophase quality. This work provides fundamental insights into regulating the development of mesophase pitch at the molecular level and offers theoretical guidance for the value-oriented conversion of FDO and the controllable preparation of high-performance mesophase pitch.

## 2. Materials and Methods

### 2.1. Raw Materials

The FDO used in this study was provided by Sinopec Refinery Sales Co., Ltd. (Shanghai, China). The general information on its physical properties and chemical composition is summarized in Table 1. The commercial mesophase pitch TMP was purchased from a pitch company, and its basic properties are listed in Table S1.

### 2.2. Fractionation of Narrow Fractions

Using a real boiling point distillation apparatus with a theoretical plate number of 15, FDO was subjected to vacuum distillation fractionation according to the ASTM D2892-20 standard method [19]. The entire fractionation process was carried out under a vacuum pressure of 1 mmHg. After fractionation, three narrow fractions with atmospheric boiling points ranging from 410 to 500 °C were selectively collected. According to the boiling point from low to high, these fractions were designated as FDO-1 (410 °C~440 °C, light fraction), FDO-2 (440 °C~470 °C, medium fraction), and FDO-3 (470 °C~500 °C, heavy fraction). The lighter fractions below 410 °C and the heavier bottom components above 500 °C were not included in the focus of this study due to their low yield or unsuitable molecular structure as precursors for high-quality mesophase pitch.

### 2.3. Preparation of Mesophase Pitch

**Figure 1:**
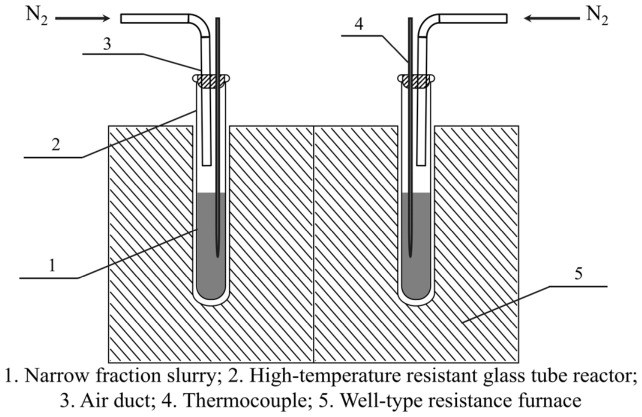
Schematic diagram of the thermal conversion device.

The thermal polycondensation experiments for mesophase preparation were conducted on a multi-tube well-type heating furnace, and the schematic diagram of the device structure is shown in Figure 1. Approximately 50 g of oil slurry raw material was weighed and placed into a high-temperature-resistant glass tube, which was then inserted into the furnace. The mixture was heated from room temperature to 440 °C at a rate of 5 °C/min in an atmospheric nitrogen atmosphere, and the reaction was held isothermally for a certain period of time. After the reaction, the glass tube was taken out and cooled to room temperature to obtain the mesophase pitch product. Depending on the different narrow fraction used as the raw material, the resulting samples were designated as MP-x-t, where x denotes the type of narrow fraction and t represents the reaction time. Thermal polycondensation experiments for each narrow fraction and each reaction time were conducted in triplicate using separately prepared sample batches. For each replicate, the key characterization parameters, including elemental composition, product yield, softening point, and anisotropic content, were measured. The results presented in this study are the average values of the three replicates. The relative deviations among the three replicates for all key parameters were consistently below five percent, confirming satisfactory reproducibility.

### 2.4. Preparation of Carbon Fibers

The melt spinning of mesophase pitch was conducted on a single filament melt spinning device [20,21] (length/diameter 3:1, diameter of 200 μm) at a temperature of 30 °C higher than the softening point of the mesophase pitch. Under a nitrogen atmosphere at 0.4 MPa, the pitch fibers were obtained at a winding speed of 240 m/min. The obtained pitch fibers were preoxidized in the air atmosphere at 280 °C for 1 h with a heating rate of 0.5 °C/min. Subsequently, the oxidized fibers were carbonized in argon at 1500 °C for 10 min. The fiber derived from pitch MP-3-12 was labeled as MPCF-3-12.

### 2.5. Characterization

The carbon residue content of the oil samples was determined using the NMC-210 (NORMALAB S.A., Paris, France) fully automatic micro carbon residue tester in accordance with ASTM D4530 [22], and the ash content was determined according to ISO 6245:2001 [23]. The SARA (saturates, aromatics, resins, and pitchenes) fractions of the slurry components were separated and quantified using liquid chromatography UPLC H-Class/xevo G2-S TOF (Waters Corporation, Milford, MA, USA) to characterize the hydrocarbon composition of the slurry. The density of the slurry was measured at 20 °C using a precision densitometer (Anton Paar GmbH, Graz, Austria) according to ASTM D4052-16 standard [24]. The kinematic viscosity of the slurry was determined at 100 °C using a capillary viscometer (Cannon Instrument Company, State College, PA, USA) according to ISO 3104:2020 standard [25]. The elemental compositions (C, H, S, N) of the slurry and mesophase products were analyzed using a Varil EL-3 elemental analyzer (Elementar, Frankfurt, Germany). The hydrocarbon composition analysis of the slurry was analyzed by Agilent 8890B-5977B gas chromatography-mass spectrometry instrument (Agilent Technologies, Santa Clara, CA, USA), following ASTM D2786 [26] and ASTM D3239 standards [27]. The toluene-insoluble (TI) content was determined according to ASTM D4312 [28], and the quinoline-insoluble (QI) content was measured following ASTM D2318 [29]. The softening point (SP) of the synthesized mesophase pitch was measured using a Dropping/Softening Point Apparatus DP90 (Mettler-Toledo, Greifensee, Switzerland). Softening points were measured three times for each sample using independently prepared sample cups, and the average value is reported. Based on the three replicates, the standard deviation was within ±1 °C for all samples. The optical texture of the mesophase pitch was observed using 506color polarizing microscope (Zeiss, Oberkochen, Germany), and the anisotropy content was quantified using ImageJ software version 1.54f (National Institutes of Health, Bethesda, MD, USA) [30]. For each mesophase pitch sample, ten representative optical micrographs were taken from randomly selected, non-overlapping fields under identical polarized light conditions. Three independent sample blocks were prepared per sample, giving a total of 30 images per sample. Each image was converted to 8-bit grayscale, and a threshold was applied using the Otsu auto-thresholding method to separate anisotropic areas from isotropic areas. The anisotropic area fraction of each image was calculated, and the reported value is the mean of the 30 measurements. The classification of mesophase optical texture is provided in Table S2. The chemical functional groups in FDO-1, FDO-2, FDO-3, and their synthesized mesophase pitches were characterized using a Thermo Fisher Nicolet 6700 Fourier transform infrared spectrometer (Thermo Fisher Scientific Inc., Waltham, MA, USA), with a wavenumber range of 4000 cm^−1^ to 400 cm^−1^. The ^1^H NMR and ^13^C NMR spectra of the slurry and mesophase pitch were measured using DMX-500 nuclear magnetic resonance spectrometer (Bruker Corporation, Billerica, MA, USA), with tetramethylsilane (TMS) and deuterated chloroform (CDCl_3_) as the standard sample and solvent, respectively, for further analysis of the chemical structure and functional groups of the samples. According to the allocation in Tables S4 and S5, MestReNova version 14.2.0 (Mestrelab Research S.L., Santiago de Compostela, Spain) is used to integrate chemical shifts in different hydrogen and carbon types. A fifth-order polynomial baseline correction was applied to the full 0–200 ppm range, with baseline points manually selected from signal-free regions. Each sample was measured three times using independently prepared solutions, and the average was taken [31]. The crystal structure of the sample was characterized using an X-ray diffractometer SmartLab 9 kW (Rigaku Corporation, Tokyo, Japan), with an X-ray wavelength of 0.15406 nm and a scanning range of 5~70°. The (002) peak of each mesophase pitch sample was fitted using two Gaussian functions in OriginPro (version 2021). The γ band (disordered carbon) and the π band (graphitic carbon) were fitted simultaneously. The Raman spectra of mesophase pitch were performed on a Raman spectrometer (HORIBA Scientific, Kyoto, Japan) with the wavenumber ranging from 500 to 2500 cm^−1^. The Raman spectra in the 1000~1800 cm^−1^ range were fitted with five Lorentzian peaks corresponding to D1 (~1350 cm^−1^), D2 (~1620 cm^−1^), D3 (~1500 cm^−1^), D4 (~1200 cm^−1^), and G (~1580 cm^−1^). MALDI-TOF MS analysis was performed on a Autoflex Speed TOF/TOF mass spectrometer (Bruker Corporation, Billerica, MA, USA) operated in positive ion reflectron mode. The matrix used was 7,7,8,8-tetracyanoquinodimethane (TCNQ, ≥98%, Sigma-Aldrich, St. Louis, MO, USA). A solvent-free sample preparation method was adopted: the pitch sample and TCNQ were combined in a 1:1 mass ratio and ground together in an agate mortar for 5 min to form a fine homogeneous powder. The laser wavelength was 355 nm. For each sample, the laser power was manually adjusted at the start of the measurement to the minimum level that produced a stable ion signal without visible fragmentation. The typical laser energy was maintained between 40% and 60% of the maximum rated output, with the exact value optimised for each sample to balance signal intensity and resolution. Each sample was measured in triplicate using independently prepared target spots; and the reported *M*_n_ and *M*_w_ values are the averages of three replicate measurements. The mass spectra were acquired by accumulating 500 laser shots per spot. The rheological properties of mesophase pitch were tested under nitrogen atmosphere using MCR302e rotational rheometer (Anton Paar, Graz, Austria). The surface morphology of carbon fibers was observed using a ZEISS Sigma 360 field emission scanning electron microscope from Germany. The fiber diameter was measured using a fiber fineness analyzer (XGD-1A, China New Fiber Instrument Co., Ltd., Shanghai, China) according to GB/T 3364-2008 [32]. For each type of carbon fiber, 20 monofilaments were measured. The diameter values were recorded as the average of 20 repeated measurements. Tensile strength and Young‘s modulus of the carbon fibres were determined following ASTM D3379 [33] using a single-filament tensile tester (YG001A, Taicang Instrument Company, Taicang, China). For each mesophase pitch material, three independent melt spinning runs were conducted under identical conditions: spinning temperature 30 °C above the respective softening point, nitrogen pressure 0.4 MPa, and winding speed 240 m/min. From each spinning run, 20 monofilaments were randomly selected and tested, resulting in a total of 60 monofilaments per material. The reported tensile strength and Young‘s modulus values are the averages across the three independent runs, with standard deviations reflecting run-to-run variability.

## 3. Results and Discussion

### 3.1. Structural and Composition of Narrow Fractions

Table 2 summarizes the fundamental properties of three narrow fraction slurries. As shown, the carbon content of all three fractions is approximately 90 wt%. As the fraction becomes heavier, the H/C ratio increases progressively, indicating that the aromatic molecules in the heavier fractions may possess a lower degree of condensation and a greater abundance of aliphatic structures. The sulfur and nitrogen contents also increase with fraction heaviness, as most of these heteroatoms are present in heavy aromatic heterocycles. Apparently, the distinct properties of the three narrow fractions can be attributed to their different distillation cut temperatures. The molecular weight of planar aromatic rings increases with boiling point, so the heaviest fraction, FDO-3, has the highest carbon residue and molecular weight.

**Figure 2:**
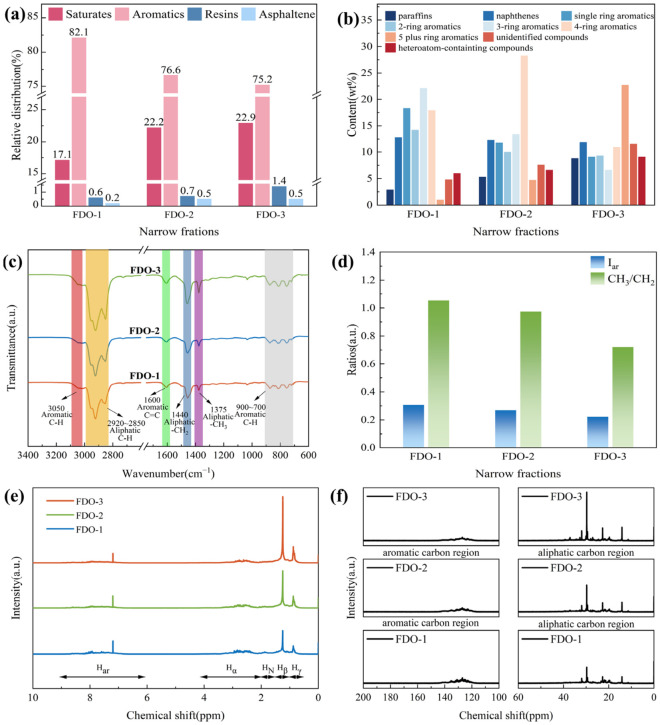
(**a**) Four-component composition; (**b**) Hydrocarbon composition; (**c**) FT-IR spectrum; (**d**) *I*_ar_ content and CH_3_/CH_2_ ratio; (**e**) ^1^H NMR spectrum; (**f**) ^13^C NMR spectrum of three narrow fractions.

SARA analysis serves as a fundamental approach for evaluating the molecular composition of heavy oils, aiming to assess their potential as precursors for mesophase pitch. Figure 2a presents the SARA composition of three narrow fraction slurries. The four-component analysis indicates that all three narrow fractions contain a high proportion of aromatics alongside relatively low contents of resins and pitchenes, which satisfies the requirements for mesophase pitch preparation. In terms of component distribution, there is an apparent negative correlation between the contents of saturates and aromatics in the narrow fractions. Specifically, FDO-1 exhibits the lowest saturates content at merely 17.1 wt%, whereas FDO-3 possesses the most abundant saturates at 22.9 wt%. Correspondingly, the aromatics content demonstrates a declining trend with increasing fraction boiling point, decreasing from 82.1 wt% in FDO-1 to 75.2 wt% in FDO-3. This finding confirms that the light fraction is relatively deficient in aliphatic hydrocarbon structures, while the heavy fraction is enriched with more aliphatic components. GC-MS (Gas Chromatography-Mass Spectrometry) was used to further identify the hydrocarbon composition information of the narrow fractions, and the results are shown in Figure 2b. Based on the distribution of identified aromatic hydrocarbons, the total aromatic content in all three fractions exceeds 75 wt%. Specifically, FDO-1 is dominated by three-ring aromatics (22.1%), followed by four-ring aromatics (17.9%). As the fraction becomes heavier, the three-ring aromatic content in FDO-2 decreases to 13.4%, while the four-ring aromatics increase substantially to 28.3%, becoming the predominant component. In FDO-3, the contents of both three-ring and four-ring aromatics are relatively reduced, whereas five-ring aromatic content increases to 22.7% and becomes the dominant component. The above distribution characteristics clearly reflect that as the boiling point of the fractions increases, the distribution of aromatic ring numbers shifts towards higher ring numbers, and the molecular size gradually increases. In addition, the content of naphthenes in the three fractions is relatively close, while the paraffin content exhibits an increasing trend following the order FDO-1 (2.9%) < FDO-2 (5.3%) < FDO-3 (8.8%). This provides further evidence that heavier fractions contain higher aliphatic hydrocarbon contents, which is in good agreement with the variation pattern of saturates content observed in the SARA analysis.

The organic functional groups of three narrow fraction slurries were characterized using FT-IR spectroscopy to further analyze the differences in their molecular structures. The results are shown in Figure 2c. All three narrow fractions exhibit similar characteristic absorption peaks, including the aliphatic and aromatic C–H stretching vibration region (2800~3100 cm^−1^), the aromatic carbon skeleton C=C stretching vibration region (1450~1650 cm^−1^), and the aromatic C–H out-of-plane bending vibration region (700~900 cm^−1^). This indicates that all three fractions are predominantly composed of polycyclic aromatic hydrocarbons with aliphatic side chains. By comparing the absorption intensities at 3050 cm^−1^ and within the 900–700 cm^−1^ region, which correspond to aromatic C–H structures, together with the intensity at 1600 cm^−1^ attributed to C=C bonds in aromatic rings, it can be observed that as the fraction becomes heavier, the signal associated with the aromatic structure gradually increases, indicating that the heavy fraction contains a more abundant polycyclic aromatic hydrocarbon component. Meanwhile, the absorption intensities representing aliphatic C–H stretching vibration (2960–2850 cm^−1^) and bending vibration (1440–1375 cm^−1^) follow the order FDO-3 > FDO-2 > FDO-1, indicating that FDO-3 possesses the highest content of aliphatic structures, which is consistent with the SARA analysis results. To further analyze the relative proportions of aromatic and aliphatic structures, curve fitting was performed on the FT-IR spectra of the three narrow fractions in the 3100~2800 cm^−1^ region. The different assignments of functional groups and corresponding wavenumbers are summarized in Table S3, and the curve fitted results are shown in Figure S1. Based on the fitted results, the aromaticity index *I*_ar_ and chain length index CH_3_/CH_2_ were calculated according to Equations (S1) and (S2), and the results are shown in Figure 2d. As illustrated, the CH_3_/CH_2_ ratio decreases significantly with increasing fraction heaviness, from 1.053 for FDO-1 to 0.721 for FDO-3. This indicates that the higher-boiling-point fractions contain longer aliphatic side chains, a higher proportion of methylene structures, and more developed branching, which aligns with trends reported in the literature [15]. Meanwhile, the *I*_ar_ value tends to decrease as the fraction becomes heavier, indicating that the content of saturated hydrocarbons increases relatively as the boiling point rises, and the proportion of C–H bonds in the aromatic structure decreases. The observation that the heavy fraction displays stronger signals in the aromatic region implies a higher degree of aromatic ring condensation in FDO-3. Although the number of aromatic C–H bonds per structural unit decreases, the total number of aromatic ring skeletons and the extent of condensation increase significantly, resulting in a stronger aromatic signal.

^1^H NMR and ^13^C NMR can analyze the distribution of hydrogen and carbon atom types in heavy oils, facilitating the inference of the structure of the narrow fractions. The assignments and corresponding chemical shifts in various types of hydrogen and carbon are listed in Tables S4 and S5, respectively, while the ^1^H NMR and ^13^C NMR spectra of the narrow fractions are presented in Figure 2e,f. By integrating the characteristic peaks at different chemical shifts, the distribution and content of hydrogen and carbon in each fraction are obtained, as listed in Table 3 and Table 4. From Table 3, it can be observed that from light fraction to heavy fraction, the content of H_ar_ and H_α_ gradually decrease, while the content of H_β_ and H_γ_ gradually increase, and the content of H_N_ remains relatively close. This trend indicates that heavier fractions contain a higher proportion of alkane components and exhibit a significant increase in the average length of alkyl side chains. Combined with the carbon atom distribution in Table 4, it is evident that the content of aliphatic carbons C_α2_, CH_2_, and CH_3_ is low in the light fraction, while the content of aromatic carbons C_ar2_ and C_ar3_ is higher than in heavier fractions. This result is highly consistent with the conclusions drawn from FT-IR and ^1^H NMR analysis. The C_ar3_/C_ar2_ ratio was compared between peri-condensed carbons and cata-condensed carbons, increasing from 0.70 in FDO-1 to 0.75 in FDO-3. This increase indicates that as the boiling range increases, the aromatic ring system becomes more compact and has the highest degree of condensation. In summary, the higher content of H_ar_ and H_α_ and lower content of H_β_ and H_γ_ in FDO-1 confirm the presence of abundant short alkyl side chains. Conversely, the lower content of H_ar_ and H_α_ and higher content of H_β_ and H_γ_ in FDO-3 reflect the highest aliphatic hydrocarbon content attributed to long alkyl side chains. The number and length of alkyl side chains in FDO-2 are intermediate between the other two fractions.

**Figure 3:**
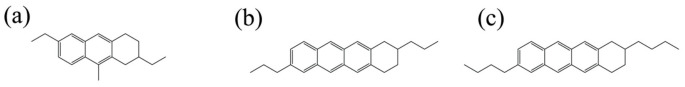
Average molecular structure model of three narrow fractions: (**a**) FDO-1; (**b**) FDO-2; (**c**) FDO-3.

In order to better illustrate the structural differences among the three narrow fraction slurries, combined with the elemental analysis and molecular weight results in Table 2, the average molecular structure parameters of FDO-1, FDO-2, and FDO-3 were calculated using the Brown–Lander method [34,35] and summarized in Table 5. The average molecular structure parameters and their calculation Equations are shown in Table S6. Table 5 shows that heavier narrow fractions possess higher total numbers of carbon atoms (*C*_T_) and hydrogen atoms (*H*_T_), which is attributed to the increased abundance of higher-molecular-weight components in these fractions. Meanwhile, compared with FDO-1 and FDO-2, FDO-3 exhibits lower aromaticity (*f*_A_), higher levels of saturated carbon (*C*_s_) and alkyl carbon (*C*_p_), indicating its higher aliphatic hydrocarbon content. Combined with the lowest aromatic ring substitution rate *σ* (0.381) and the longest average molecular chain length *L*(4) for FDO-3, it indicates that FDO-3 has fewer alkyl side chains but the longest length. In addition, the aromatic ring condensation degree (H_Au_/C_A_) of the three narrow fractions all exceed 0.5, indicating that their molecular structures are characteristically cata-condensed [36,37]. Based on the average molecular structural parameters in Table 5, schematic models of the average molecular structures for the three narrow fractions were constructed, as shown in Figure 3. The calculated structural parameters and inferred molecular structure jointly demonstrate that FDO-1 possesses the highest aromatic structure content and abundant short alkyl side chains. FDO-2 exhibits a slight reduction in side chain quantity accompanied by increased molecular size and side chain length. In contrast, FDO-3 is characterized by the highest aliphatic structure content, the fewest aromatic substituents, and the longest side chain length.

### 3.2. Properties and Structure of Mesophase Pitch

The elemental composition and yield of the mesophase pitch products obtained from the thermal polycondensation of the three narrow fractions at varying durations are presented in Table 6, with the yields calculated according to Equation (S4). Compared with the feedstocks, the mesophase pitch products exhibit lower H/C atomic ratios, which are attributable to the dehydrogenation reactions occurring during thermal polycondensation [38]. As polycondensation proceeds, more hydrogen and light components are eliminated. Consequently, for products derived from the same narrow fraction, both the H/C ratio and yield progressively decrease, indicating that extended reaction time results in a higher degree of condensation. Notably, under the same polycondensation time (12 h), the H/C ratios and yields of MP-1-12, MP-2-12, and MP-3-12 gradually decrease with increasing fraction heaviness, suggesting that the degree of aromatic condensation in the resulting product increases with the heaviness of the narrow fraction. Meanwhile, the sulfur and nitrogen contents of the products derived from each narrow fraction exhibit a decreasing trend with prolonged polycondensation time.

Table 6 also presents the TI and QI contents of all mesophase pitch samples. Both TI and QI increase with longer polycondensation time and with a higher boiling range of the parent narrow fraction. This trend indicates progressive condensation of aromatic molecules into larger, less soluble polycyclic structures. For MP-1-16 derived from FDO-1, TI reaches 49.68 wt% while QI is only 30.53 wt%, indicating that most components are insoluble in toluene but remain soluble in quinoline. In contrast, MP-3-12 derived from FDO-3 exhibits the highest values among all samples, with a TI of 71.76 wt% and a QI of 54.87 wt%. The FDO-2-derived products show intermediate rates of increase in TI and QI. These differences reflect the lower condensation degree of FDO-1-derived products, whereas FDO-3-derived products achieve the highest condensation degree at 12 h, consistent with the observed trends in their H/C ratios.

**Figure 4:**
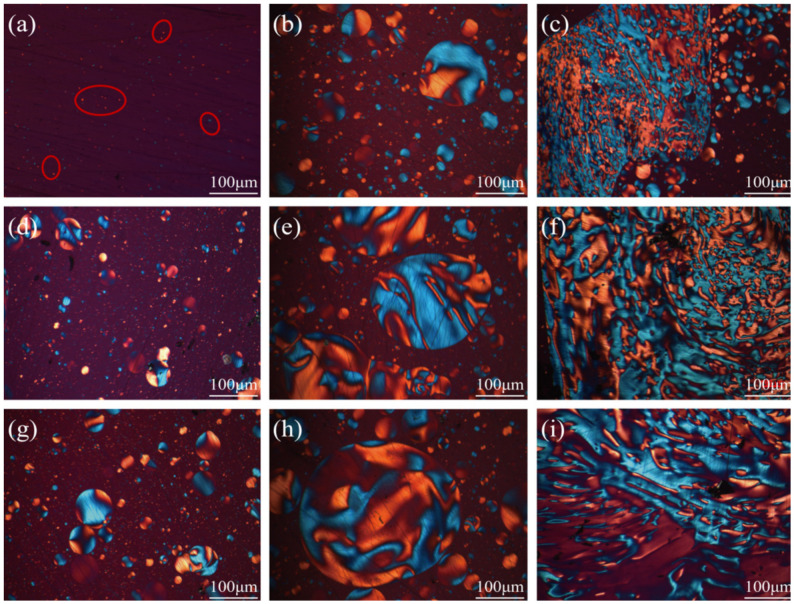
Polarized-light micrographs of mesophase pitch products: (**a**) MP-1-12; (**b**) Mp-1-14; (**c**) Mp-1-16; (**d**) Mp-2-10; (**e**) Mp-2-12; (**f**) Mp-2-14; (**g**) MP-3-8; (**h**) Mp-3-10; (**i**) Mp-3-12.

**Figure 5:**
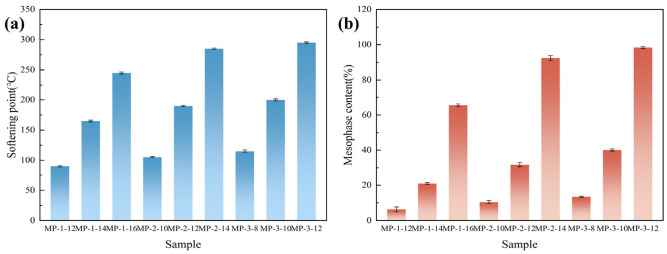
The softening point and anisotropic content of mesophase pitch products: (**a**) Softening point of mesophase pitch; (**b**) Anisotropic content of mesophase pitch.

The typical polarized light micrographs of the mesophase pitch products obtained from the three narrow fractions at different thermal polycondensation times are presented in Figure 4, while the corresponding softening points and anisotropic contents are shown in Figure 5. As evident from Figure 4 and Figure 5, both the softening point and anisotropic content of the products derived from all three narrow fractions increase significantly with prolonged reaction time. Comparative analysis of the textural evolution within products from the same narrow fraction reveals distinct polycondensation behavior. For MP-1-12 (Figure 4a), the polarised light micrograph shows a predominantly isotropic dark matrix with a small number of isolated, brightly coloured mesophase spheres (indicated by red circles). No continuous anisotropic domains are observed at this stage. This indicates the formation of mesophase spheres that have not fully developed, exhibiting relatively small dimensions. Upon extending polycondensation to 14 h (Figure 4b), the mesophase spheres undergo substantial growth, with diameters ranging from approximately 5 μm to 150 μm. Concurrently, the approach, contact, and coalescence of spheres are observed, suggesting that the growth and coalescence of spheres occurred synchronously. When the condensation time was extended to 16 h (Figure 4c), the large spheres formed through coalescence of numerous smaller ones experienced large-scale disintegration due to excessive volume, where surface tension becomes insufficient to maintain the spherical morphology. The resulting optical texture is predominantly characterized by fine mosaic structures, with some inadequately developed small mesophase spheres still present. This phenomenon is attributed to the relatively low reactivity of FDO-1, which results in insufficient growth of some spheres, coupled with the limited availability of aliphatic hydrocarbon components and restricted volatile release, ultimately leading to the formation of mosaic textures. The textural evolution of products derived from FDO-2 and FDO-3 similarly follows the sequence of sphere nucleation, growth, coalescence, and disintegration. MP-2-10 (Figure 4d) displays mesophase spheres with dimensions ranging from 5 μm to 60 μm. Upon polycondensation for 12 h (Figure 4e), the sphere size range expands to 5–200 μm. Further extending the reaction to 14 h (Figure 4f) results in the development of a continuous coarse mosaic texture. For MP-3-8 (Figure 4g), spherical textures with sizes ranging from 5 μm to 120 μm are observable. After polycondensation for 10 h (Figure 4h), the product exhibits large mesophase spheres reaching dimensions of 5–300 μm. At 12 h (Figure 4i), the texture evolves into a small-domain flow texture measuring approximately 40 × 100 μm.

The above texture differences are due to the different molecular structures of narrow fractions in the raw materials. FDO-1 possesses the lowest aliphatic hydrocarbon content and the shortest alkyl side chains. Consequently, the amount of light components volatilized during thermal polycondensation is limited, making it difficult to form an effective directional gas flow within the system. Due to the lack of shear-induced orientation, aromatic macromolecules are mainly stacked in a random manner, with poor molecular orientation, ultimately forming a fine mosaic texture. In contrast, FDO-2 exhibits increased aliphatic hydrocarbon content and longer alkyl side chains, resulting in increased volatilization of light components during thermal polycondensation. A directional gas flow of a certain intensity begins to form within the system, exerting an initial shear-inducing orientation on aromatic macromolecules. Simultaneously, the moderate cracking of aliphatic side chains supplies additional hydrogen radicals to the system, promoting aromatic ring condensation reactions and enabling the orderly growth of the mesophase. However, as both the intensity and duration of the gas flow remain insufficient to drive long-range ordered molecular alignment, the aromatic lamellae achieve orientation only within limited regions, forming a coarse mosaic texture larger than FDO-1 but not yet developed into a continuous flow domain. The mosaic textures, stemming from feedstocks with high aromaticity and insufficient aliphatic structures, are prone to generating defects during subsequent carbonization, which adversely affect the mechanical properties of carbon fibers [31,39]. By contrast, flow-domain textures exhibit more ordered structures and represent the ideal precursor texture morphology for the preparation of high-performance carbon fibers.

By comparing the optical texture of the three narrow fractions over time and combining the softening point and anisotropy content in Figure 5, it was found that the softening point (295.1 °C) and anisotropy content (98.4%) of MP-3-12 were significantly higher than those of MP-1-12 (90.0 °C, 6.3%) and MP-2-12 (190.0 °C, 31.7%) under the same polycondensation time (12 h), indicating that its raw material FDO-3 has higher thermal reactivity and undergoes a greater extent of polycondensation within the same time. Further observation of the evolution rate of the properties of each narrow fraction over time showed that after extending the polycondensation time by 4 h, the anisotropy content of MP-3 increased the most, which further confirmed the conclusion that FDO-3 has the highest reactivity. High-boiling fractions contain greater quantities of heavy components such as polycyclic aromatic hydrocarbons and pitches. These components are more prone to dehydrogenative condensation reactions during thermal polycondensation, generating free radical small molecules [13] and thereby conferring enhanced thermal reactivity. As previously mentioned, FDO-3 possesses the highest content of heavy components, which accounts for its strongest thermal reactivity. Notably, the abundant alkanes and naphthenes present in FDO-3 also serve as favorable components for liquid-phase carbonization, functioning as natural hydrogen donors during pyrolysis [40]. MP-3-12 exhibits a moderately elevated softening point of 295 °C and an anisotropic content of 98.8%. This combination of high anisotropy and a moderately high softening point is consistent with literature reports on high-quality mesophase pitches [4,41]. Despite the elevated softening point, the abundant aliphatic components in the parent fraction FDO-3 play a critical role in preserving fluidity during polycondensation. The alkyl side chains and naphthenic structures effectively maintain a favourable rheological environment, facilitating the ordered arrangement of aromatic macromolecules and enabling near-complete anisotropy [31].

**Figure 6:**
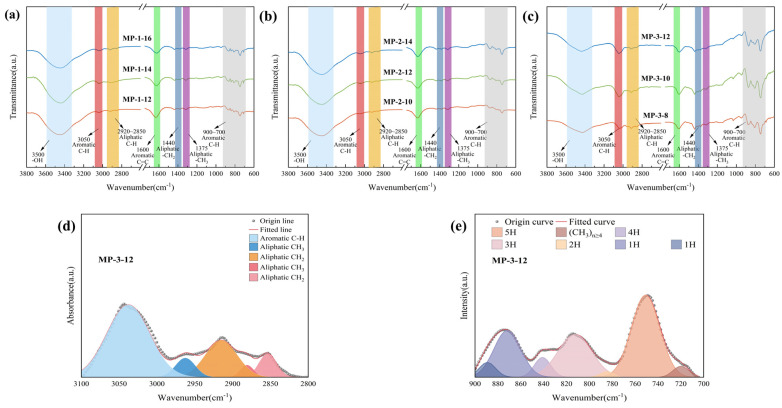
(**a**) FT-IR spectra of MP-1; (**b**) FT-IR spectra of MP-2; (**c**) FT-IR spectra of MP-3; (**d**) Fitted images of infrared spectra of MP-3-12 at 3100~2800 cm^−1^; (**e**) Fitted images of infrared spectra of MP-3-12 at 900~700 cm^−1^.

The FT-IR spectra presented in Figure 6a–c provide further insight into the molecular structures of the mesophase pitches derived from the three narrow fractions at different times. As shown in the figures, the spectra of each mesophase pitch exhibit similar characteristic absorption peaks: the aromatic C–H stretching vibration at 3050 cm^−1^, the aromatic C=C skeletal vibration at 1600 cm^−1^, and the aromatic C–H out-of-plane bending vibrations in the 900 cm^−1^~700 cm^−1^ region, confirming that the main structures are all aromatic compounds. Meanwhile, the absorption peaks in the range of 2950 cm^−1^~2850 cm^−1^ correspond to the stretching vibrations of aliphatic –CH_2_ and –CH_3_ compounds, while the absorption peaks near 1450 cm^−1^ and 1380 cm^−1^ belong to the bending vibrations of aliphatic –CH_2_ and –CH_3_ compounds. Compared to the aromatic structures, the intensity of the aliphatic absorption peaks is significantly weaker, indicating that the mesophases derived from the three narrow fractions are all composed of highly condensed aromatic rings with low aliphatic side chain content. Additionally, the broad absorption peak observed at 3550~3200 cm^−1^ is assigned to –OH structure. Compared with the FT-IR spectra of their respective narrow fraction feedstocks, the intensities of the aliphatic absorption peaks at 2920 cm^−1^~2850 cm^−1^ and 1440 cm^−1^~1375 cm^−1^ of each mesophase pitch are reduced, indicating that aliphatic hydrocarbon cracking occurred during the thermal polycondensation process. This trend becomes increasingly pronounced with prolonged polycondensation time. A comparison of the mesophase pitches prepared from the three narrow fractions at the same polycondensation times reveals that the relative intensities of the aromatic absorption peaks at 3050 cm^−1^ and 1600 cm^−1^ follow the order MP-1 < MP-2 < MP-3, demonstrating that MP-3 possesses the highest aromaticity. This observation is consistent with the trend in H/C ratio variation in elemental analysis.

Peak fitting was performed on the FT-IR spectra of the mesophase pitches in the ranges of 3100 cm^−1^~2800 cm^−1^ and 900 cm^−1^~700 cm^−1^ to further analyze the evolution of functional groups. The structural parameters calculated based on the fitted results from Equations (S1)–(S3) are listed in Table 7. The fitted results for MP-3-12 are shown in Figure 6d,e. The fitted results for the other samples are shown in Figures S2 and S3.

As shown in Table 7, when the polycondensation time was 12 h, the *I*_ar_ value of the resulting mesophase pitch products increased sequentially with increasing narrow fraction heaviness, with MP-1-12, MP-2-12, and MP-3-12 exhibiting values of 0.646, 0.749, and 0.945, respectively. This indicates that heavier fractions yield products with lower alkyl structure content and consequently higher aromaticity, which is consistent with the H/C ratio variation in elemental analysis. Simultaneously, the CH_3_/CH_2_ ratio increased significantly with increasing fraction heaviness, rising from 0.834 for MP-1-12 to 3.783 for MP-3-12, indicating that the alkyl side chains in the product gradually shortened. Combined with the decrease in CH_3_/CH_2_ of the feedstock fractions with increasing heaviness in Figure 2d, this observation suggests that the heavy fraction undergoes more extensive cracking of alkyl structures during polycondensation, reflecting its higher reactivity. This conclusion is consistent with the findings derived from softening point and anisotropic content evolution. Alkyl side chains are prone to breakage during thermal condensation to generate primary free radicals, which are strong reactive sites in the reaction system. Therefore, the heavy fraction with the highest content of long alkyl side chains has the highest concentration of reactive sites. The size of aromatic lamellae can be characterized by *I*_os_, where larger *I*_os_ values indicate a greater tendency for aromatic molecules to adopt linear arrangements via ortho-substitution rather than forming aromatic lamellae through peri-condensation [14]. In Table 7, *I*_os_ is in the order of MP-1-12 (0.538) > MP-2-12 (0.487) > MP-3-12 (0.445), indicating that MP-3-12 has the highest aromaticity and the largest molecular dimension. Further investigation into the influence of polymerization time on the structural parameters of the same narrow fraction product revealed that *I*_ar_ and CH_3_/CH_2_ gradually increased with increasing time, while *I*_os_ showed an opposite trend. This is because prolonged polymerization time promotes deeper condensation of aromatic rings, resulting in continuous growth of aromatic lamellae, accompanied by the sustained cracking and release of aliphatic side chains from the system.

**Figure 7:**
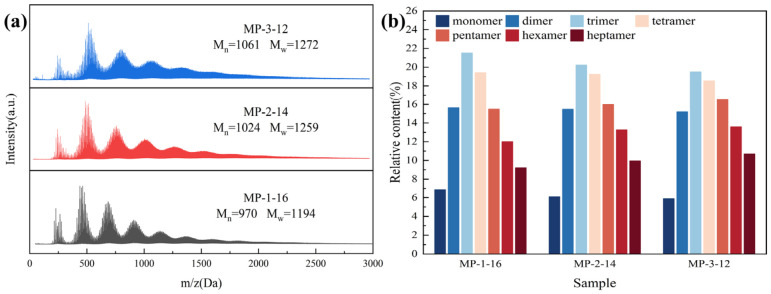
(**a**) MALDI-TOF MS spectra of MP-1-16, MP-2-14 and MP-3-12; (**b**) Molecular weight distribution of MP-1-16, MP-2-14 and MP-3-12.

The molecular weight distribution of the mesophase pitches obtained from the three narrow fractions was characterized by MALDI-TOF MS. The mass spectra of MP-1-16, MP-2-14 and MP-3-12 are shown in Figure 7a. The mass-to-charge ratio (m/z) of the three mesophase pitches ranged from 0 to 2000, showing a continuous distribution from monomers to heptamers. Compared with their respective feedstocks, the mesophase pitch products show significantly increased molecular weights. The number-average molecular weights of MP-1-16, MP-2-14, and MP-3-12 were calculated using Equations (S5) and (S6) to be approximately 965 Da, 1010 Da, and 1055 Da, respectively, with a relative standard deviation of about 5% based on triplicate measurements. These values indicate that the three narrow fractions underwent significant polycondensation during thermal cracking. To further elucidate the evolution of molecular weight during polycondensation, the oligomers were categorized according to their molecular weight intervals, and the relative content of each component was determined by calculating the peak area proportions [42], as shown in Figure 7b. It is evident that trimers constitute the most abundant component in all three mesophase pitch samples. The relative contents of monomers to tetramers showed a decreasing trend in the order MP-1-16 > MP-2-14 > MP-3-12, while the relative contents of pentamers to heptamers increased in the opposite order. This result indicates that the formation of mesophase pitch is a dynamic process of simultaneous oligomerization of multiple components. While oligomers continuously condense to form mesomers, mesomers also continuously condense to form polymers. Therefore, multiple polymers from low to high molecular weight always coexist in the system. As the degree of condensation increases, the proportion of oligomers gradually diminishes, while the proportion of polymers correspondingly increases, leading to an overall elevation in molecular weight. In Figure 7b, the sample with a larger *M*_n_ has a higher proportion of pentamers and higher polymers, further verifying this rule.

**Figure 8:**
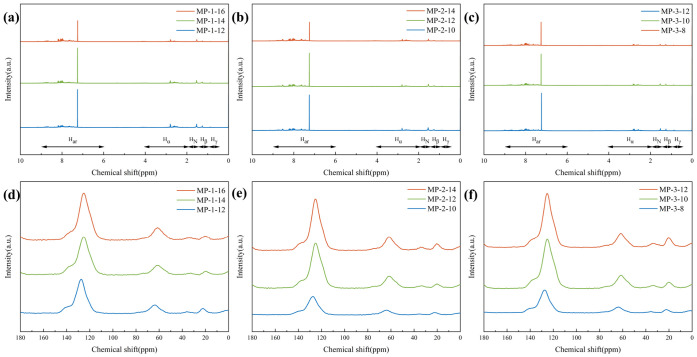
(**a**) ^1^H NMR spectrum of MP-1; (**b**) ^1^H NMR spectrum of MP-2; (**c**) ^1^H NMR spectrum of MP-3; (**d**) ^13^C NMR spectrum of MP-1; (**e**) ^13^C NMR spectrum of MP-2; (**f**) ^13^C NMR spectrum of MP-3.

Mesophase pitches prepared from three narrow fraction slurries were characterized by ^1^H NMR and ^13^C NMR spectra to compare the evolution of molecular structure before and after the reaction, as shown in Figure 8. Based on the ^1^H NMR spectra in Figure 8a–c, the various hydrogen types in each mesophase pitch were normalized and integrated, and the corresponding structural parameters were calculated, with the results summarized in Table 8. Compared with their respective feedstocks, all mesophase pitches showed a trend of increasing aromatic hydrogen (H_ar_) content and decreasing aliphatic hydrogen (H_α_, H_β_, H_γ_, and H_N_) content, which also confirms the occurrence of alkyl side chain cleavage, cycloalkanes dehydrogenation, and aromatic ring condensation during thermal polycondensation. For products derived from the same narrow fraction, the H_ar_ content progressively increases with prolonged polycondensation time. Under identical polycondensation time, the products obtained from heavier fractions display substantially higher H_ar_ contents than those from lighter fractions, following the order MP-3-12 (77.59) > MP-2-12 (71.36) > MP-1-12 (66.47). Contrary to the trend of H_ar_ content change, the contents of H_α_, H_β_, H_γ_, and H_N_ decreased with the extension of polycondensation time and the increase in fraction heaviness, indicating that the long alkyl side chain decomposes into short alkyl side chains through cracking, cyclization and aromatization during thermal polycondensation, and escapes in the form of small-molecule gases such as hydrogen and methane, allowing the aromatic ring to undergo further oligomerization and facilitating mesophase formation [43]. MP-3-12 has the least number of aliphatic substituents and shorter alkyl side chains, which is consistent with the pattern of FT-IR parameter changes in Table 7, indicating that its condensation and cracking reactions are the deepest, allowing FDO-3, which has the longest alkyl side chain in the raw material, to achieve sufficient chain breakage and removal after condensation [44].

**Figure 9:**
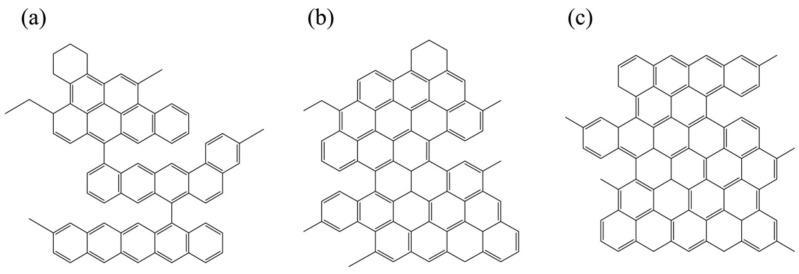
Average molecular structure models of (**a**) MP-1-16; (**b**) MP-2-14; (**c**) MP-3-12.

The molecular structures of the products derived from the three narrow-fraction slurries during liquid-phase carbonization were further elucidated using the Brown–Lander method [34,35]. The average molecular structure parameters of MP-1-16, MP-2-14, and MP-3-12 were calculated and summarized in Table 9. As shown in the table, the aroma index (*f*_a_) increases in the order of MP-1-16 (0.883) < MP-2-14 (0.903) < MP-3-12 (0.935), while the condensation index (H_AU_/C_A_) shows the opposite trend, indicating that MP-3-12 has the highest degree of condensation and aromaticity. The aromatic ring condensation index H_Au_/C_A_, which theoretically distinguishes cata-condensed (H_Au_/C_A_ > 0.5) from peri-condensed (H_Au_/C_A_ < 0.5) structures, shows a gradual decrease from MP-1-16 (0.519) to MP-2-14 (0.474) to MP-3-12 (0.448), reflecting a gradual increase in the proportion of peri-condensed aromatic units as the feedstock boiling range increases. For MP-1-16, the value of 0.519 is only marginally above 0.5. Given the inherent uncertainty of the Brown–Lander method, this value lies near the boundary, suggesting that the aromatic structure of MP-1-16 contains both cata- and peri-condensed domains. The progressive decrease from MP-1-16 to MP-3-12 indicates a continuous shift toward a more peri-condensed architecture in the heavy-fraction product. The substitution rate (*σ*) gradually decreased in the order of MP-1-16 (0.117) > MP-2-14 (0.095) > MP-3-12 (0.074), indicating that MP-3-12 had the lowest aliphatic substituent content, a trend consistent with the FT-IR analysis results. Based on the average molecular structure parameters in Table 9, molecular structure models of the three mesophase pitches were constructed, as shown in Figure 9. MP-2-14 and MP-3-12, with their high aromatic carbon ratios and forced condensation modes, indicate that their molecular skeletons are mainly composed of aromatic rings fused along a two-dimensional direction. Their fewer and shorter aliphatic side chains result in less intermolecular steric hindrance, allowing the polycyclic macromolecules to approximately maintain a planar configuration rather than being distorted by the steric effects of aliphatic substituents. Therefore, their molecular structure models are disk-shaped, with MP-3-12 exhibiting the highest planarity, having the lowest H_AU_/C_A_ value and substitution rate. Conversely, the cata-condensed structure of MP-1-16 causes torsion at the methylene bridge connections, disrupting the overall planarity of the molecule and resulting in a wrinkled lamellar configuration. In summary, the molecular structure of MP-1-16 is predominantly composed of cata-condensed aromatic rings with a small number of long alkyl side chains, while MP-2-14 and MP-3-12 mainly consist of highly condensed planar aromatic lamellae with few short alkyl side chains.

The ^13^C NMR spectra of each mesophase pitch products are listed in Figure 8d–f. The fitted curves after fitting the spectra based on the chemical shifts in the characteristic peaks are shown in Figure S4. The distribution of characteristic carbon peaks [45,46] is listed in Table 10. The molecular average carbon skeleton parameters in the ^13^C NMR spectra are quantitatively analyzed by fitting. As shown in Table 10, compared with their respective feedstocks, all mesophase pitch samples exhibit significantly reduced aliphatic carbon (C_al_) content alongside substantially increased aromatic carbon (C_ar_) content, indicating the formation of macromolecular condensed polycyclic aromatic structures through polycondensation reactions of the small-molecule aromatics present in the feedstocks [39]. With prolonged condensation time and increasing fraction heaviness, the C_ar_ content shows a steady increase, from 81.52% of MP-1-12 to 89.59% of MP-3-12, while the C_al_ content correspondingly decreases from 18.38% to 10.41%. Within the C_al_, the CH_3_ and CH_2_, representing methyl and methylene groups, also decreased with increasing polymerization time and fraction heaviness, indicating a continuous reduction in aliphatic side chains, which is consistent with the analysis results of FT-IR and ^1^H NMR. C_α2_ represents the methylene carbon between two aromatic rings, showing the similar trend as CH_3_ and CH_2_, suggesting that the proportion of aromatic structures connected via cata-condensation decreased and the degree of condensation increased. Within the C_ar_, C_ar3_ represents peri-condensed aromatic carbons where the aromatic structures adopt a planar arrangement, while C_ar2_ denotes aromatic carbon with aromatic substituents, whose structure is mainly arranged linearly. The condensation type of mesophase pitch can be determined by calculating C_ar3_/C_ar2_ [47]. A higher C_ar3_/C_ar2_ ratio suggests that aromatic carbons are predominantly arranged in rigid planar configurations, whereas a lower ratio indicates the dominance of semi-rigid linear structures [48]. In Table 10, the C_ar3_/C_ar2_ values showed an increasing trend with increasing polymerization time and fraction heaviness, reaching a maximum value of 6.35 in MP-3-12. This result indicates that the proportion of aromatic carbons with substituents in polycyclic aromatic hydrocarbon macromolecules gradually decreases, and the molecular configuration changes from cata-condensation to peri-condensation, which is consistent with the variation pattern of H_AU_/C_A_ in Table 9.

**Figure 10:**
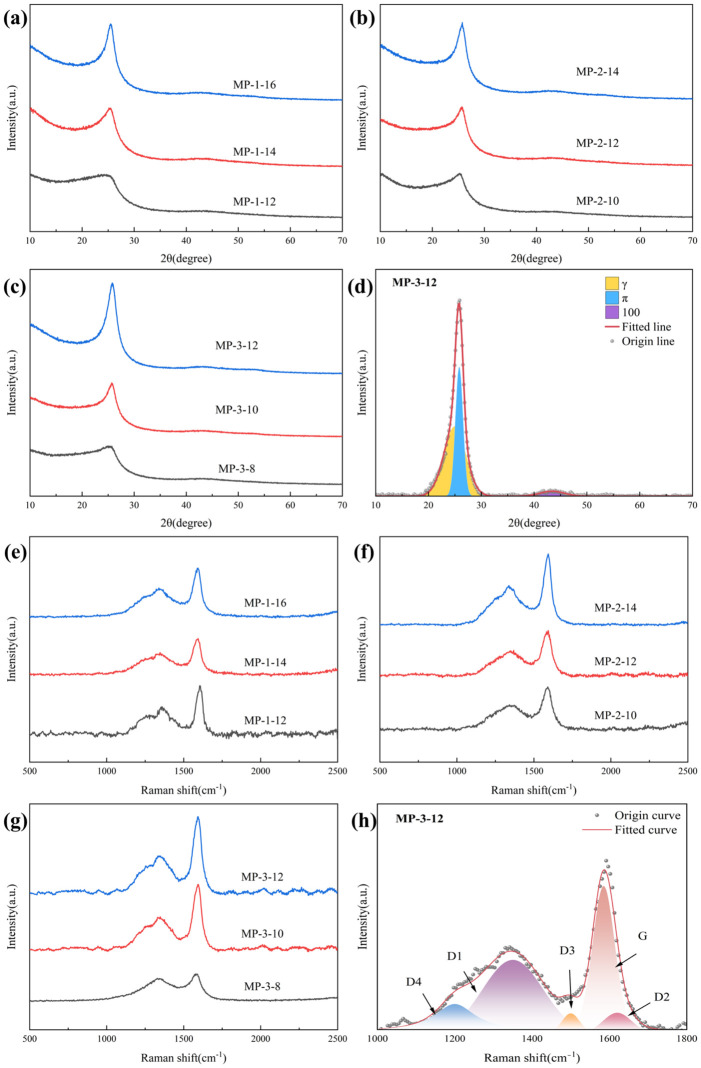
XRD spectra of (**a**) MP-1; (**b**) MP-2; (**c**) MP-3; (**d**) Fitted (002) peak region of MP-3-12 using a two-Gaussian model; Raman spectra of (**e**) MP-1; (**f**) MP-2; (**g**) MP-3; (**h**) Fitted Raman spectrum of MP-3-12 using a five-Lorentzian peak model.

The evolution of the microcrystalline structure of mesophase pitches was investigated by XRD and peak fitting. The XRD spectra of the products obtained from each narrow fraction at different polycondensation times are shown in Figure 10a–c. All samples exhibit a (002) diffraction peak at 2*θ* = 23~25° and a (100) peak at 2*θ* = 43~44°. With prolonged polycondensation time, the width of the (002) peak progressively decreases, while the peak shifts toward higher 2*θ* values and its intensity increases, indicating a significant enhancement in the structural order of the products [49] and gradual optimization of the graphite microcrystalline structure. The asymmetric (002) peak was fitted as a *π* band representing graphite carbon and a *γ* band representing disordered carbon [50,51]. The curve fitted result of MP-3-12 is shown in Figure 10d, and the fitted curves of the other samples are shown in Figure S5. As shown in Figure S5, with the extension of polycondensation time and the increase in fraction heaviness, the *γ* band exhibits narrowing of the peak width, reduction in peak height, and a marked decrease in integrated area, indicating a progressive decline in the proportion of disordered carbon within the microcrystalline structure. Based on the peak position and half width at half maximum (FWHM) obtained by the fitted curve, the interlayer spacing (*d*_002_), stacking height (*L*_c_), and number of stacked layers (*N*) of all samples were calculated by Equation (S7)–(S9), and the results are listed in Table 11. For mesophase pitches derived from the same narrow fraction, *d*_002_ gradually decreases with prolonged polycondensation time, while *L*_c_ and *N* continuously increase. Shimanoe [52] proposed that the significant anisotropic structure can only be obtained when *N* is at least greater than eight layers. This argument can be mutually verified by the observation that the *N* of MP-1-12 is only 7.432 nm and its isotropic texture shown in Figure 4a. The above results show that extending the polycondensation time promotes the growth and ordered stacking of aromatic lamellae, facilitating the progressive evolution from an initially disordered isotropic state to an ordered anisotropic structure. At a constant polycondensation time of 12 h, as the fraction becomes heavier, the *d*_002_ of the mesophase products decreases markedly, while both *L*_c_ and *N* increase substantially. This indicates that the mesophase pitches derived from heavier fractions exhibit faster growth of graphite microcrystallites and higher structural order. This phenomenon is primarily attributed to the abundant long alkyl side chains in the heavy fraction, which release substantial quantities of small molecular volatiles during thermal cracking. The directional escape of these volatiles generates a shear-induced orientation effect on the aromatic lamellae, promoting ordered stacking of planar molecules along the gas flow direction, enhancing intermolecular interactions, and ultimately leading to the formation of graphite-like microcrystalline structures with higher long-range order. In summary, both extending the polymerization time and utilizing heavier fractions can effectively improve the ordered arrangement of aromatic carbon layers and optimize the graphite microcrystalline structure, with the improvement effect of fraction heaviness being more significant. This conclusion is highly consistent with the optical texture evolution and the FTIR and NMR analysis results.

The Raman spectra of mesophase pitches obtained from three narrow fractions at different polycondensation times are shown in Figure 10e–h. All samples showed two characteristic peaks at 1350 cm^−1^ and 1580 cm^−1^, corresponding to the D band representing disordered structure and the G band representing ordered graphite structure, respectively [53,54]. With prolonged polycondensation time, the full width at half maximum (FWHM) of the D band of each fraction gradually decreased and the peak shape became narrower, indicating that extending the polycondensation time can effectively reduce structural defects in mesophase pitch. To further quantitatively analyze the types of carbon microcrystalline structures, curve fitting of the Raman spectra was performed. The distribution of each characteristic peak and the corresponding vibration mode are shown in Table S8 [55,56]. The Raman spectrum fitted results are shown in Figure S6, and the peak fitted results of MP-3-12 are shown in Figure 10h. The degree of order of the carbon layer can be quantitatively compared by the graphite microcrystal content (*A*_G_/*A*_All_) and the content of various defective carbon microcrystal (*A*_Di_/*A*_G_) [57]. The results are shown in Table 11. For mesophase pitches derived from the same narrow fraction, the ratios of *A*_D1_/*A*_G_, *A*_D2_/*A*_G_, and *A*_D3_/*A*_G_ all decreased with increasing polymerization time, while *A*_G_/*A*_All_ gradually increased, consistent with the qualitative analysis results of Raman spectroscopy. Comparison of products derived from different fractions revealed that the *A*_D1_/*A*_G_, *A*_D2_/*A*_G_, and *A*_D3_/*A*_G_ ratios of the mesophase pitches obtained from the heavy fraction were generally significantly lower than those from the light fractions and medium fractions, while *A*_G_/*A*_All_ increased with increasing fraction heaviness. This trend was most pronounced for products obtained at a constant polycondensation time of 12 h. The decrease in *A*_D1_/*A*_G_ indicates a reduction in defects at the edges of aromatic lamellae, mainly related to the cleavage and removal of aliphatic side chains. As polycondensation time extends and fraction heaviness increases, the degree of alkyl side chain cracking intensifies, leading to reduced steric hindrance effects. The decrease in *A*_D2_/*A*_G_ reflects improved regularity in the vertical stacking of aromatic lamellae. Both prolonged polycondensation time and increased fraction heaviness effectively reduces *A*_D2_/*A*_G_, promoting tighter stacking of lamellae in the three-dimensional direction. Simultaneously, the decrease in *A*_D3_/*A*_G_ further confirms a decreased content of amorphous carbon within the system, with the microcrystalline structure approaching that of ideal graphite crystals. MP-3-12 exhibits the highest *A*_G_/*A*_All_ and the lowest *A*_D1_/*A*_G_, *A*_D2_/*A*_G_ and *A*_D3_/*A*_G_, indicating the highest content of ideal graphite microcrystals, the fewest structural defects, and the optimal degree of order. Combined FT-IR and NMR analyses reveal that the mesophase pitch derived from the heavy fraction has a higher degree of condensation, shorter aliphatic side chains, and superior molecular planarity, all of which facilitate the ordered arrangement of aromatic lamellae. In summary, the structural order of mesophase pitch improves with both prolonged polycondensation time and increased fraction heaviness, a trend that is in strong agreement with the findings from XRD analysis.

### 3.3. Development Mechanism of Narrow Fraction Liquid-Phase Carbonization

**Figure 11:**
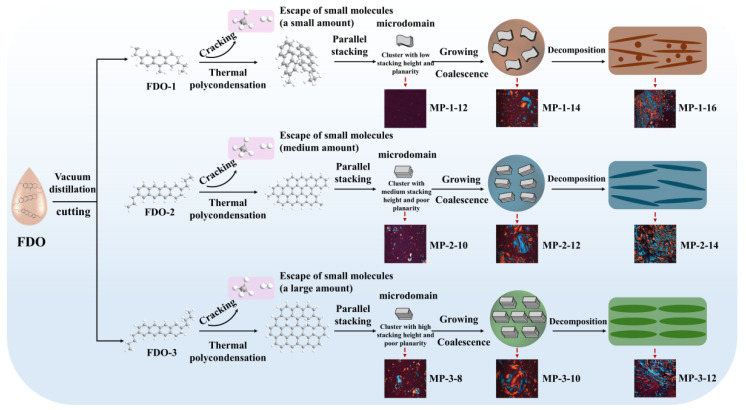
Schematic diagram of possible transformation mechanisms in three narrow fraction thermal condensation processes.

Based on the above analysis, the schematic mechanism of the thermal polycondensation and self-assembly process of the three narrow fractions with the development of polycondensation time is proposed, as shown in Figure 11. In the early stage of the reaction, the slurry molecules undergo intense thermal cracking, where weak covalent bonds in the alkyl side chains cleave to generate free radicals [58,59,60]. Concurrently, small molecules produced from cracking, including naphthenes, mono- and di-aromatic hydrocarbons, and light hydrocarbons, volatilize and escape from the reaction system [1,61,62]. Free radical fragments undergo coupling and dehydrogenation reactions [63], with aromatic radicals recombining through mutual coupling to form oligomers such as dimers and trimers, thereby preliminarily constructing planar aromatic macromolecular layers. The planar aromatic molecular layers stack on each other to form microdomains, which are manifested as mesophase spherical textures under an optical microscope. As the reaction progresses, polycondensation and aromatization reactions become dominant within the system. Oligomers further combine with each other to form larger planar macromolecular polycyclic aromatic hydrocarbons, such as hexamers and heptamers. The increased molecular weight enhances interlayer interactions, leading to tighter stacking of the aromatic macromolecular layers. With the continuous increase in both the size and quantity of mesophase spheres, the spheres collide with each other, merge and aggregate, and further merge with other spheres. The sphere size continues to increase until the surface tension reaches a critical value, at which point the mesophase spheres lose their ability to maintain a spherical morphology and disintegrate and break down [64], forming continuous domain textures, as exemplified by MP-3-12. As condensation proceeds, the mesophase domains continuously develop, accompanied by progressive enhancements in molecular planarity, aromatic condensation degree, and the ordering of aromatic carbon layers, while the melt viscosity also continues to rise. The melt viscosity evolution during this process is critically dependent on the alkyl side chain characteristics of each narrow fraction. Although FDO-1 and FDO-2 exhibit lower initial viscosities than FDO-3 due to their smaller molecular weights and shorter alkyl chains, their limited aliphatic content proves insufficient to maintain a low viscosity regime under high-temperature polycondensation. As the reaction progresses, cross-linking and condensation of aromatic rings cause a steep increase in melt viscosity. This viscosity surge impedes the mobility and coalescence of mesophase spheres, restricts the ordered stacking of aromatic lamellae, and ultimately leads to the formation of a poorly developed mosaic texture. In contrast, FDO-3 possesses the highest initial viscosity because of its larger molecular size and longer alkyl side chains. During thermal cracking, its abundant aliphatic structures release a continuous stream of small-molecule volatiles. Based on the observed structural trends, we hypothesize that the directional escape of these volatiles may create a local shear field. For a non-Newtonian fluid such as the reacting pitch, such a shear field could in principle induce alignment of the polycyclic aromatic molecules along the flow direction, a phenomenon known as shear thinning, thereby lowering local melt viscosity and promoting ordered stacking of aromatic lamellae. [65,66]. This proposed mechanism is consistent with the observed correlation between the heavy fraction and the development of a well-ordered flow texture. The heavy fraction exhibits the highest volatile release potential, as indicated by its higher aliphatic content and the steepest yield reduction in Table 6. However, direct quantitative validation, such as measurements of gas evolution rate, volatile flux, or in situ flow visualization, is not available in the present study. Therefore, the interpretation shown in Figure 11 should be regarded as a hypothesis requiring further experimental confirmation.

Thus, although all three narrow fractions follow a radical-mediated conversion mechanism during thermal polycondensation [67], the differences in their molecular structure lead to distinct system environments during liquid-phase carbonization, which in turn determine the derived molecular size, configuration, and ultimately the optical texture. For the light fraction FDO-1, the abundance of aliphatic side chains facilitates the formation of methylene bridge structures between aromatic rings, resulting in poor planarity of the aromatic lamellae that hinders self-stacking and leads to low stacking heights of mesophase clusters. Additionally, the shortest aliphatic side chains and lowest aliphatic hydrocarbon content yield the lowest concentration of free radicals in the system and poor reactivity, making it difficult to achieve products with high anisotropic content even after 16 h of reaction. For the middle fraction FDO-2, the number of aliphatic side chains is reduced compared to FDO-1, and the association mode transitions from methylene bridges to phenyl bridges, enhancing both planarity and stacking height. The increased total aliphatic hydrocarbon content elevates the free radical concentration, improving reactivity and enabling an anisotropic content of 92.5% after 14 h of polycondensation. However, the limited volatilization of light components during liquid-phase carbonization fails to generate sufficient gas flow to induce shear orientation among the multiple mesophase clusters, resulting in the formation of mosaic optical texture. In contrast, the heavy fraction FDO-3 possesses the fewest aliphatic side chains but the longest chain length, with the highest proportion of highly condensed phenyl bridge structures, forming high-quality clusters characterized by large stacking heights and small interlayer spacings. Furthermore, its highest reactivity enables an anisotropic content of 98% after only 12 h of polycondensation. Critically, the cracking of long aliphatic side chains generates small molecular gases that escape the system, forming a sustained directional gas flow. This flow exerts a shear-induced orientation effect on the aromatic macromolecular layers, promoting their alignment along the gas flow direction and thereby facilitating the formation of domain optical texture.

### 3.4. Evaluation of Spinning Performance

**Figure 12:**
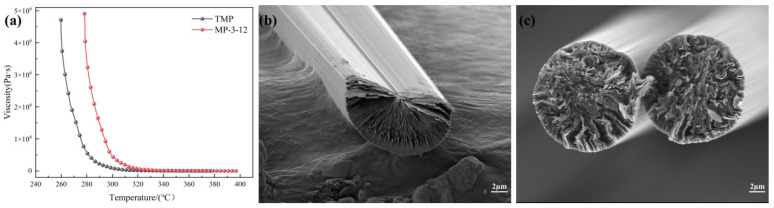
(**a**) Viscosity–temperature curve of TMP and MP-3-12; (**b**) Carbon fiber cross-sectional image of TMP; (**c**) Carbon fiber cross-sectional image of MP-3-12.

MP-3-12 and a commercial mesophase pitch (TMP) were selected for the preparation of mesophase pitch-based carbon fibers through melt spinning, pre-oxidation, and carbonization. The spinnability of the mesophase pitches was evaluated using a rotational rheometer. As shown in Figure 12a, TMP exhibits a relatively lower average viscosity and higher fluidity. The melt spinning results for both pitches are summarized in Table 12. Although the continuous spinning duration of MPCF-3-12 is slightly shorter than that of TMPCF, its fiber diameter is significantly smaller, indicating superior overall spinnability. Figure 12b,c present the fiber cross-sectional images of TMPCF and MPCF-3-12, and additional micrographs are provided in Figure S7 of the Supporting Information. TMPCF displays a radial arrangement of graphite microcrystallites, whereas MPCF-3-12 exhibits a radially folded structure. A pronounced split of approximately 158° is observed in the TMPCF cross-section, forming a typical “pac-man” structure. This phenomenon arises from the high degree of order in its aromatic lamellae, where the highly oriented radial arrangement is subjected to concentrated circumferential stress during carbonization, leading to fiber splitting. In contrast, MPCF-3-12 exhibits a regular circular cross-section with good uniformity. The presence of an appropriate amount of aliphatic side chains in the molecular structure of its precursor, MP-3-12, facilitates gradual cracking during carbonization. The release of small molecular gases alleviates the accumulation of internal stress, effectively mitigating the formation of split structures [68] and resulting in a highly ordered graphite structure with fewer defects. The mechanical properties of the resulting carbon fibers were tested, with the results presented in Table 13. Due to the split structure, TMPCF exhibits relatively low tensile strength and Young’s modulus. In contrast, MPCF-3-12 benefits from the highly ordered planar aromatic macromolecules and an appropriate amount of alkyl side chains in its precursor, which significantly reduce structural defects during carbonization. Consequently, MPCF-3-12 exhibits both high strength and high modulus. As shown in Table 13, while MPCF-3-12 does not match the tensile strength of the highest-performance commercial mesophase pitch-based carbon fibre products, such as Dialead K13D2U at 3.7 GPa or Granoc XN-90 at 3.4 GPa, those commercial products are graphitized at ultra-high temperatures and derived from naphthalene-based synthetic pitches with highly optimised processing conditions. Considering that MPCF-3-12 was prepared from a single narrow fraction of FCC slurry oil without any optimisation of spinning, stabilisation, or carbonisation conditions, its mechanical properties, with an average tensile strength of approximately 1.45 GPa, are noteworthy and fall within the moderate-to-high range for laboratory-prepared pitch-based carbon fibres derived from petroleum residues (Table 13). This study confirms that precise control of fractionation and mesophase pitch molecular structure is an effective approach for the preparation of high-performance carbon fibres, and further optimisation of processing parameters could potentially close the gap with commercial benchmarks.

### 3.5. Practical and Economic Considerations for Industrial Application of the Heavy Fraction

From an industrial perspective, the adoption of the heavy narrow fraction FDO-3 as a mesophase pitch precursor entails a series of practical considerations regarding feedstock utilization, distillation cost, and product value. Vacuum distillation of FDO slurry typically allows the recovery of approximately 9.15 wt% of material within this high-boiling range. The remainder of the slurry, comprising lighter fractions with boiling points below 470 °C, is not rendered obsolete. On the contrary, direct recycling of such lighter aromatic streams to the feed of an RFCC unit has been demonstrated as a technically viable practice that converts these materials into high-yield gasoline and light olefins without compromising process efficiency [72]. Therefore, the fractionation step improves the utilization of raw materials, and the relatively small but ideal fraction of the slurry is transferred to high-value mesophase pitch production, while the majority continues to be used for traditional refining.

The technical feasibility of obtaining narrow boiling cuts from FCC decant oil by vacuum distillation has been extensively documented. Feng [73] successfully separated the oil extracted from catalytic cracking slurry into eight narrow fractions with boiling point intervals of 20 °C. They distilled these fractions using a standard vacuum distillation apparatus and systematically analyzed the aromatic composition and structural evolution of these fractions. Similarly, vacuum distillation has been widely used to separate ethylene tar into light and heavy fractions, with heavy fractions showing superior ability to form mesophase pitch [13,15]. Narrow fraction cutting relies on routine adjustments to key parameters such as distillation cutting point and reflux ratio, which are completely within the design flexibility range of traditional atmospheric and vacuum distillation towers, and the cost is within a controllable range. It is crucial that the substantial improvement in product performance justifies the moderate cost increase associated with narrow-cut vacuum distillation. Direct carbonization of the whole FCC slurry typically yields a mesophase pitch with an anisotropic content below 90%, which is difficult to process by continuous melt spinning. In contrast, MP-3-12 derived from FDO-3 exhibits excellent spinnability, achieving an anisotropic content of 98.8% and a softening point of 295 °C. Moreover, the carbon fibers prepared from this mesophase pitch possess mechanical properties superior to those derived from commercial pitch. In high-value applications such as aerospace components and electronic devices, product performance dictates economic feasibility. Therefore, the outstanding quality of the final carbon material more than compensates for the additional cost incurred by narrow-cut vacuum distillation. This analysis confirms that the targeted utilization of well-defined narrow fractions is both technically effective and economically reasonable for the production of high-performance carbon materials.

## 4. Conclusions

This study systematically investigated the thermal polycondensation behavior, mesophase formation pathways, and resulting structural properties of three narrow fractions derived from FDO. The results demonstrate that the molecular structural characteristics of the narrow fractions, particularly the length and content of alkyl side chains, are the key factors governing their thermal reactivity and mesophase evolution pathways. The light fraction, possessing the shortest alkyl side chains and the lowest content, exhibits weak thermal reactivity and a slow reaction progression. The insufficient degree of aromatic ring condensation results in products retaining extensive isotropic regions and dispersed spheres, making it difficult to form a continuous anisotropic texture. The middle fraction, with increased alkyl side chain length and content, shows enhanced thermal reactivity and undergoes a complete mesophase evolution process. However, due to the limited volatile light components within the system and the absence of effective shear-induced orientation, a mosaic texture is ultimately formed, with restricted structural order. The heavy fraction, characterized by the longest alkyl side chains and the highest content, releases abundant free radicals and small-molecule volatiles during thermal cracking. The directional escape of these volatiles generates a shear-induced effect on the aromatic lamellae, accelerating their rapid growth and ordered stacking. Under conditions of 440 °C for 12 h, this fraction yields high-quality mesophase pitch featuring a small-domain flow texture, low softening point, and a mesophase content exceeding 98%. The derived carbon fibers exhibit mechanical properties exceeding those of carbon fibers prepared from a commercial reference pitch (TMP) processed under identical laboratory conditions. In conclusion, the heavy fraction serves as an ideal precursor for the preparation of mesophase pitch with high anisotropy and excellent spinnability. This study not only provides a feasible pathway for the value-added directional conversion of FDO but also offers theoretical support for the molecular-level design of high-performance mesophase pitch.

## Figures and Tables

**Table 1:** Physical and chemical properties of FDO slurry.

Properties		FDO
Density (20 °C)/g·cm^−3^		1.06
Micro carbon residues/wt.%		0.11
Ash content/wt.%		0.01
Viscosity (100 °C)/mm^2^·s^−1^		12.66
Number average molecular weight (*M*_n_)		293
Elemental composition/wt.%	C	90.36
	H	8.78
	S	0.45
	N	0.18
	H/C	1.17
Four components (SARA) analysis/wt.%	Saturates	20.09
	Aromatics	74.26
	Resins	4.79
	Asphaltene	0.86

**Table 2:** Physical and chemical properties of three types of three narrow fractions.

Properties		FDO-1	FDO-2	FDO-3
Elemental composition/wt.%	C	91.04	90.68	89.96
	H	8.95	9.15	9.88
	S	0.44	0.5	0.57
	N	0.18	0.19	0.2
	H/C	1.18	1.21	1.32
Density (20 °C)/g·cm^−3^		1.06	1.05	1.03
Micro carbon residues/wt.%		0.01	0.05	1.08
Ash content/wt.%		0.01	0.01	0.01
Viscosity (100 °C)/mm^2^·s^−1^		10.09	17.76	30.27
Number average molecular weight (*M*_n_)		246	292	337

**Table 3:** Hydrogen distribution in narrow fraction slurries.

Properties		FDO-1	FDO-2	FDO-3
Hydrogen distribution	H_ar_ (wt.%)	24.26	20.85	16.32
	H_α_ (wt.%)	32.59	27.88	21.08
	H_N_ (wt.%)	8.85	8.71	8.59
	H_β_ (wt.%)	23.84	29.22	37.75
	H_γ_ (wt.%)	10.55	13.40	16.34

**Table 4:** Carbon distribution in cutting narrow fraction slurries.

Samples	C_ar2_ (wt.%)	C_ar3_ (wt.%)	C_α2_ (wt.%)	CH_2_ (wt.%)	CH_3_ (wt.%)	C_ar3_/C_ar2_
FDO-1	38.23	26.88	8.11	16.84	10.03	0.70
FDO-2	35.4	25.76	9.42	19.48	9.98	0.73
FDO-3	32.16	24.23	11.07	22.91	9.92	0.75

**Table 5:** The average molecular structure parameters of FDO-1, FDO-2 and FDO-3.

Parameters	FDO-1	FDO-2	FDO-3
*f*_A_	0.608	0.575	0.507
H_Au_/C_A_	0.782	0.741	0.715
*σ*	0.402	0.394	0.381
*C*_T_	19	22	25
*H*_T_	22	27	33
*C*_A_	11	13	13
*C*_S_	7	9	12
*C*_N_	3	3	3
*C*_P_	4	6	10
*R*_T_	3	4	4
*R*_A_	2	3	3
*R*_N_	1	1	1
*L*	2	3	4

**Table 6:** Basic physicochemical properties of mesophase pitch products.

Samples	Elemental Composition/wt.%				H/C	Yield/wt.%	TI/wt.%	QI/wt.%
	C	H	S	N				
MP-1-12	93.92	5.52	0.4	0.16	0.71	66.35	23.58	2.78
MP-1-14	94.20	5.31	0.35	0.14	0.68	60.84	36.43	18.94
MP-1-16	94.53	5.02	0.31	0.14	0.64	56.52	49.68	30.53
MP-2-10	93.98	5.45	0.4	0.17	0.70	64.28	27.42	8.53
MP-2-12	94.42	5.09	0.33	0.16	0.65	58.19	42.11	23.77
MP-2-14	94.98	4.59	0.28	0.15	0.58	54.38	64.31	44.72
MP-3-8	94.09	5.36	0.37	0.18	0.68	62.31	30.58	13.55
MP-3-10	94.69	4.83	0.32	0.16	0.61	54.35	48.46	26.96
MP-3-12	95.32	4.27	0.26	0.15	0.54	43.56	71.76	54.87

**Table 7:** FTIR parameters of mesophase pitches derived from narrow fractions.

Samples	*I*_ar_	CH_3_/CH_2_	*I*_os_
MP-1-12	0.646	0.834	0.538
MP-1-14	0.724	1.011	0.492
MP-1-16	0.802	2.847	0.478
MP-2-10	0.713	0.748	0.514
MP-2-12	0.749	1.147	0.487
MP-2-14	0.821	3.038	0.476
MP-3-8	0.727	0.655	0.502
MP-3-10	0.871	1.361	0.472
MP-3-12	0.945	3.783	0.445

**Table 8:** Hydrogen distribution of mesophase pitches derived from narrow fractions.

Samples	H_ar_ (wt.%)	H_α_ (wt.%)	H_N_ (wt.%)	H_β_ (wt.%)	H_γ_ (wt.%)
MP-1-12	53.77	23.89	8.36	10.88	3.63
MP-1-14	59.68	19.84	7.91	9.52	2.86
MP-1-16	66.47	16.29	7.23	8.06	1.93
MP-2-10	55.85	22.16	8.14	10.36	3.51
MP-2-12	63.94	18.42	7.18	8.35	2.47
MP-2-14	71.36	14.49	5.94	7.26	1.54
MP-3-8	57.13	21.75	8.03	10.06	3.42
MP-3-10	66.19	16.82	6.76	7.93	2.14
MP-3-12	77.59	11.56	4.36	5.42	0.96

**Table 9:** The average molecular structure parameters of MP-1-16, MP-2-14 and MP-3-12.

Parameters	MP-1-16	MP-2-14	MP-3-12
*f*_A_	0.883	0.903	0.935
H_Au_/C_A_	0.519	0.474	0.448
*σ*	0.117	0.095	0.074
*C*_T_	74	79	83
*H*_T_	47	46	45
*C*_A_	66	71	78
*C*_S_	9	8	5
*C*_N_	4	3	0
*C*_P_	5	5	5
*R*_T_	16	22	24
*R*_A_	17	23	24
*R*_N_	1	1	0
*L*	1.348	1.227	1.039

**Table 10:** Carbon distribution of mesophase pitches derived from narrow fractions.

Samples	C_ar_ (wt.%)			C_al_ (wt.%)			C_ar3_/C_ar2_
	C_ar2_	C_ar3_	CH_ar_	C_α2_	CH_2_	CH_3_	
MP-1-12	12.66	56.22	12.64	4.32	4.78	9.38	4.44
MP-1-14	12.25	60.41	12.02	3.69	3.77	7.86	4.93
MP-1-16	11.59	63.85	12.88	2.88	3.41	5.39	5.51
MP-2-10	12.75	59.78	11.05	4.24	4.12	8.06	4.69
MP-2-12	11.42	62.27	12.12	3.42	3.59	7.18	5.45
MP-2-14	10.98	66.84	11.32	2.65	3.07	5.14	6.09
MP-3-8	12.64	61.76	10.10	3.82	3.82	7.86	4.89
MP-3-10	11.53	65.60	9.65	3.19	3.24	6.78	5.69
MP-3-12	11.13	70.67	7.79	2.51	2.87	5.03	6.35

**Table 11:** XRD and Raman parameters of mesophase pitches derived from narrow fractions.

Samples	*d*_002_ (Å)	*L*_c_ (nm)	*N*	*A*_D1_/*A*_G_	*A*_D2_/*A*_G_	*A*_D3_/*A*_G_	*A*_G_/*A*_ALL_
MP-1-12	0.3537	2.272	7.423	2.6716	0.3696	0.3055	0.2076
MP-1-14	0.3498	3.343	10.557	1.8560	0.2904	0.2515	0.2388
MP-1-16	0.3476	4.145	12.925	1.6191	0.2542	0.2314	0.2529
MP-2-10	0.3521	2.476	8.031	2.1486	0.4691	0.2735	0.219
MP-2-12	0.3471	4.047	12.659	1.6287	0.3458	0.2618	0.2561
MP-2-14	0.3458	4.973	15.379	1.3348	0.3258	0.2452	0.2877
MP-3-8	0.3512	2.857	9.135	1.7588	0.2477	0.2573	0.2285
MP-3-10	0.3462	4.391	13.683	1.3453	0.1736	0.1372	0.2833
MP-3-12	0.3448	5.174	16.006	1.0206	0.1573	0.1158	0.3461

**Table 12:** Spinnabilities of mesophase pitches and mechanical properties of pitch-based carbon fibers.

Samples	Diameter (μm)	Continuous Spinning Time (min)	Tensile Strength (GPa)	Young’s Modulus (GPa)	Samples
TMPCF	14.02 (±0.2)	28	1.10 (±0.20)	125 (±17)	TMPCF
MPCF-3-12	13.65 (±0.3)	26	1.45 (±0.32)	151 (±19)	MPCF-3-12

**Table 13:** Comparison of MPCF-3-12 with pitch-based carbon fibers reported in previous studies and commercial products.

Samples	Diameter (μm)	Tensile Strength (GPa)	References
MPCF-3-12	13.65	1.44	This work
CF-MP-100	~20.00	0.95	Ref. [4]
MPCF-PP	14.20	1.26	Ref. [31]
MPCF-12	11~13	1.39	Ref. [69]
RCNMPCF-1	14.62	1.36	Ref. [70]
MPCF-320.5	-	3.23	Ref. [71]
Mitsubishi Dialead K13D2U	~11	3.70	Mitsubishi Chemical
Nippon Graphite Fiber Granoc XN-90	~10	3.43	Nippon Graphite Fiber Corporation

## Data Availability

The original contributions presented in this study are included in the article/Supplementary Material. Further inquiries can be directed to the corresponding author.

## Supplementary Materials

The following supporting information can be downloaded at https://www.mdpi.com/article/10.3390/ma19122528/s1, Table S1: Basic properties of TMP; Table S2: Classification of the optical microstructure of MP; Table S3: Band wavenumber and assignments in the FT-IR spectra; Table S4: Chemical shifts for different types of hydrogen by NMR spectroscopy; Table S5: Chemical shifts for different types of carbon by NMR spectroscopy; Table S6: The average molecular structure parameter and meaning; Table S7: Distribution of polymers peak positions in mesophase pitch; Table S8: Band assignments of different vibration modes in Raman spectrum; Figure S1: The fitted curve of FTIR of narrow fraction slurry at 3100–2850 cm^−1^: (a) FDO-1; (b) FDO-2; (c) FDO-3; Figure S2: The fitted curve of FTIR of mesophase pitch at 3100-2850 cm^−1^: (a) MP-1-12; (b) MP-1-14; (c) MP-1-16; (d) MP-2-10; (e) MP-2-12; (f) MP-2-14; (g) MP-3-8; (h) MP-3-10; Figure S3: The fitted curve of FTIR of mesophase pitch at 900-700 cm^−1^: (a) MP-1-12; (b) MP-1-14; (c) MP-1-16; (d) MP-2-10; (e) MP-2-12; (f) MP-2-14; (g) MP-3-8; (h) MP-3-10; Figure S4: The fitted curve of ^13^C NMR of mesophase pitch: (a) MP-1-12; (b) MP-1-14; (c) MP-1-16; (d) MP-2-10; (e) MP-2-12; (f) MP-2-14; (g) MP-3-8; (h) MP-3-10; (i) MP-3-12; Figure S5: The fitted curves of XRD spectra of mesophase pitch: (a) MP-1-12; (b) MP-1-14; (c) MP-1-16; (d) MP-2-10; (e) MP-2-12; (f) MP-2-14; (g) MP-3-8; (h) MP-3-10; Figure S6: Raman spectra and fitted curves of mesophase pitch: (a) MP-1-12; (b) MP-1-14; (c) MP-1-16; (d) MP-2-10; (e) MP-2-12; (f) MP-2-14; (g) MP-3-8; (h) MP-3-10; Figure S7: Typical SEM micrographs of (a) TMPCF; (b) MPCF-3-12; Equation (S1): The aromaticity index *I*_ar_; Equation (S2): The chain length index CH_3_/CH_2_; Equation (S3): The ortho-substitution index *I*_os_; Equation (S4): The product yield; Equation (S5): The number-average molecular weight *M*_n_; Equation (S6): The weight-average molecular weight *M*_w_; Equation (S7): The interlayer spacing *d*_002_; Equation (S8): The stacking thickness *L*_c_; Equation (S9): The number of stacked layers *N*.

## Abbreviations

The following abbreviations are used in this manuscript:

FDO	Fluid Catalytic Cracking decant oil
MP	Mesophase pitch
MPCF	Mesophase pitch-based carbon fiber
GC-MS	Gas Chromatography-Mass Spectrometry
