# Supplementary material for: Study on the Structural Characteristics of Narrow Fractions of Catalytic Cracking Slurry and the Formation Pathway of Mesophase Pitch
Source: Materials (Basel). 2026 Jun 11;19(12):2528. doi: 10.3390/ma19122528 (PMC13303349; doi:10.3390/ma19122528)
Supplement: Supplementary file 1 [file materials-19-02528-s001.zip › materials-4340738-supplementary.pdf]

materials

MDPI

Article

Supplementary Information

Study on the structural characteristics of narrow fractions of catalytic cracking slurry and the formation pathway of mesophase pitch

Xuesong Shan [1,2], Shuandi Hou [1,2,*], Renqing Chu [2], Yun Wu [2], Yuanyuan Zhang [2], Dan Guo [2], Yongen Gao [2], Shiwen Li [2] and Zihui Ma [2]

[1] SINOPEC Research Institute of Petroleum Processing Co. Ltd., Beijing 100083, China; shanxuesong.ripp@sinopec.com (X.S.)

[2] SINOPEC Dalian Petrochemical Research Institute Co., Ltd., Dalian 116045, China; churenqing.fshy@sinopec.com (R.Q.); wuyun.fshy@sinopec.com (Y.W.); zhangyuanyuan.fshy@sinopec.com (Y.Z.); guodan.fshy@sinopec.com (D.G.); gaoyongen.fshy@sinopec.com (Y.G.); lishiwen.fshy@sinopec.com(S.L.); mazihui.fshy@sinopec.com (Z.M.)

* Correspondence: houshuandi.fshy@sinopec.com

Table S1. Basic properties of TMP

Sample	Element content/%					SP/℃	Ash content/%	Residue carbon/%	M_n
	C	H	S	N	H/C				
MP	95.05	4.21	0.26	0.12	0.53	283.2	0.004	88.73	598.91

SP-Softening Point;M_n-Number-average molecular weight

Academic Editor:

Received: date
Revised: date
Accepted: date
Published: date

Citation:

Copyright:

Table S2. Classification of the optical microstructure of MP.

Texture Name		Optical size
Isotropic		No optical activity (present in interphase asphalt with mesophase content <100%)
Mosaic-type	Fine-grain mosaic-type	Anisotropic radius <1.5 μm
	Medium-grain mosaic-type	Anisotropic radius in the range of 1.5 ~5 μm
	Coarse-grain mosaic-type	Anisotropic radius in the range of 5~10 μm
	Supra-mosaics	Aligned mosaics
Domain-type	Small domains	Anisotropic radius in the range of 10.0–60.0 μm
	Medium-flow anisotropy	Anisotropic length <30 μm, width <5 μm
	Coarse-flow anisotropy	Anisotropic length in the range of 30~60 μm, width in the range of 5~10 μm
	Flow domain anisotropy	Anisotropic length >60 μm, width >10 μm
Broad-type		Anisotropic radius >60 μm

$$I_{ar}=\frac{A_{3040cm^{-1}}}{A_{3040cm^{-1}}+A_{2920cm^{-1}}} \quad (S1)$$

$$CH_3/CH_2=\frac{A_{3040cm^{-1}}}{A_{2920cm^{-1}}} \quad (S2)$$

$$I_{os}=\frac{A_{750cm^{-1}}}{A_{750cm^{-1}}+A_{814cm^{-1}}+A_{840cm^{-1}}+A_{880cm^{-1}}} \quad (S3)$$

I_{ar} represents the fraction of aromatic carbons without alkyl substituents, indicating the degree of condensation of the sample.The CH_3/CH_2 ratio reflects the length of the alkyl side chains. I_{os} denotes the fraction of aromatic rings that are ortho-substituted (i.e., possessing four adjacent hydrogen atoms) relative to all aromatic rings containing at least one hydrogen atom, serving as an indicator of the relative molecular size and degree of condensation of aromatic molecules.

Table S3. Band Wavenumber and assignments in the FT-IR Spectra

**Positions (cm^{-1})**	**2953**	**2923**	**2896**	**2879**	**2853**	**895**	**876860**
Assignments	Asym. RCH_3	Asym. R_2CH_2	R_3CH	Sym. RCH_3	Sym. R_2CH_2	1H	1H 1H
Positions (cm^{-1})	838	815	790	750	742	720	695
Assignments	2H	2H	3H	4H	4H	$(CH3)_{n\geq4}$	5H

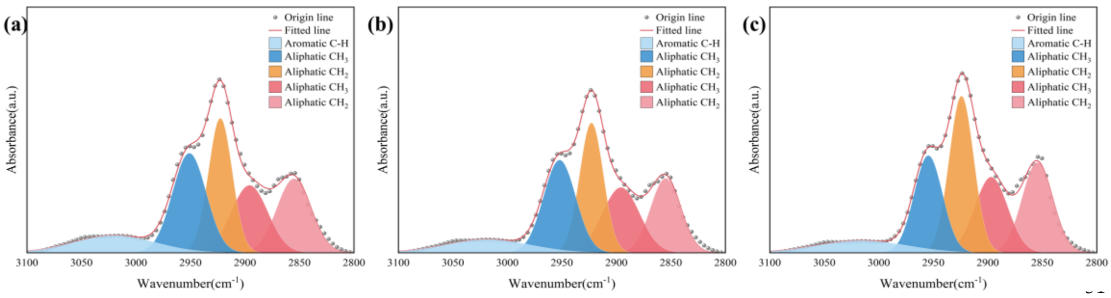

Figure S1. The fitted curve of FTIR of narrow fraction slurry at 3100-2850 cm-1: (a) FDO-1, (b) FDO-2, (c) FDO-3.

Table S4. Chemical shifts for different types of Hydrogen by NMR spectroscopy

Hydrogen type	Definition	Chemical shift(ppm)
H_{ar}	aromatic hydrogen	9.0~6.0
H_{α}	aliphatic hydrogen in methyl or methylene groups in *α* position to an aromatic ring	4.0~2.0
H_N	naphthenic hydrogen	2.0~1.5
H_{β}	aliphatic hydrogen in methyl or methylene groups in β position to an aromatic ring	1.5~1.0
H_{γ}	aliphatic hydrogen in methyl or methylene groups in γ position to an aromatic ring	1.0~0.5

Table S5. Chemical shifts for different types of carbon by NMR spectroscopy

Carbon type	Definition	Chemical shift(ppm)
C_{ar2}	catacondensed aromatic carbons, aromatic carbon with heteroatomic or aromatic substituents, and aromatic carbons joined to aliphatic chains	160.0~129.5
C_{ar3}	Pericondensed aromatic carbons and protonated aromatic carbons	129.5~108
$C_{\alpha 2}$	bridge/hydroaromatic structures	49.3~34
CH_2	all other methylene carbons	34~23
CH_3	aliphatic carbon of methyl groups	23~17

Table S6. The average molecular structure parameter and meaning

Parameters	Meaning	Formula

f_A	Fraction of aromatic carbon	$f_A=\frac{C/H-(H_\alpha+H_\beta+H_\gamma)/(2H_T)}{C/H}$
H_{Au}/C_A	Condensation of aromatic ring system	$\frac{H_{Au}}{C_A}=\frac{H_A/H_T-H_\alpha/(2H_T)}{C/H-(H_\alpha+H_\beta+H_\gamma)/(2H_T)}$
σ	Substitution index of aromatic ring system	$\sigma=\frac{H_\alpha/2}{H_A+H_\alpha/2}$
C_T	Total carbons	$C_T=\frac{M\times C\%}{12\times 100}$
H_T	Total hydrogens	$H_T=\frac{M\times H\%}{100}$
C_A	Aromatic carbons per average molecule	$C_A=C_T\times f_A$
C_S	Saturated carbons per average molecule	$C_S=C_T-C_A$
C_N	Naphthenic carbons per average molecule	$C_N=4R_N$（cata-condensed polymerization）<br>$C_N=3R_N$（peri-condensed polymerization）
C_P	Alkyl carbons per average molecule	$C_P=C_S-C_N$
R_T	Total rings	$R_T=C_T+1-C_A/2-H_T/2$
R_A	Aromatic rings per average molecule	$R_A=(C_A-2)/4$（cata-condensed polymerization）<br>$R_A=(C_A-4)/3$（peri-condensed polymerization）
R_N	Naphthenic rings per average molecule	$R_N=R_T-R_A$

L	Average chain length	$L=\frac{C_P}{N_{CH_3}}$, $N_{CH_3}=\frac{C_S}{2+N_{CH_2}/N_{CH_3}}$, $\frac{N_{CH_2}}{N_{CH_3}}=2.93\frac{A_{1460}}{A_{1380}}-3.70$

$$\text{product yield}=\frac{m_2}{m_1}$$

(S4)

In this equation, m_1 represents the mass of the feedstock slurry, expressed in grams (g); m_2 denotes the mass of the product obtained after thermal conversion, also in grams.

$$M_n=\frac{\sum_i m_i I_i}{\sum_i I_i} \quad \text{(S5)}$$

$$M_w=\frac{\sum_i m_i^2 I_i}{\sum_i m_i I_i} \quad \text{(S6)}$$

m_i: molecular mass, expressed in Da; I_i: corresponding proportion, representing the intensity of mi in the mass spectrum.expressed in %; representing the intensity of m_i in the mass spectrum.

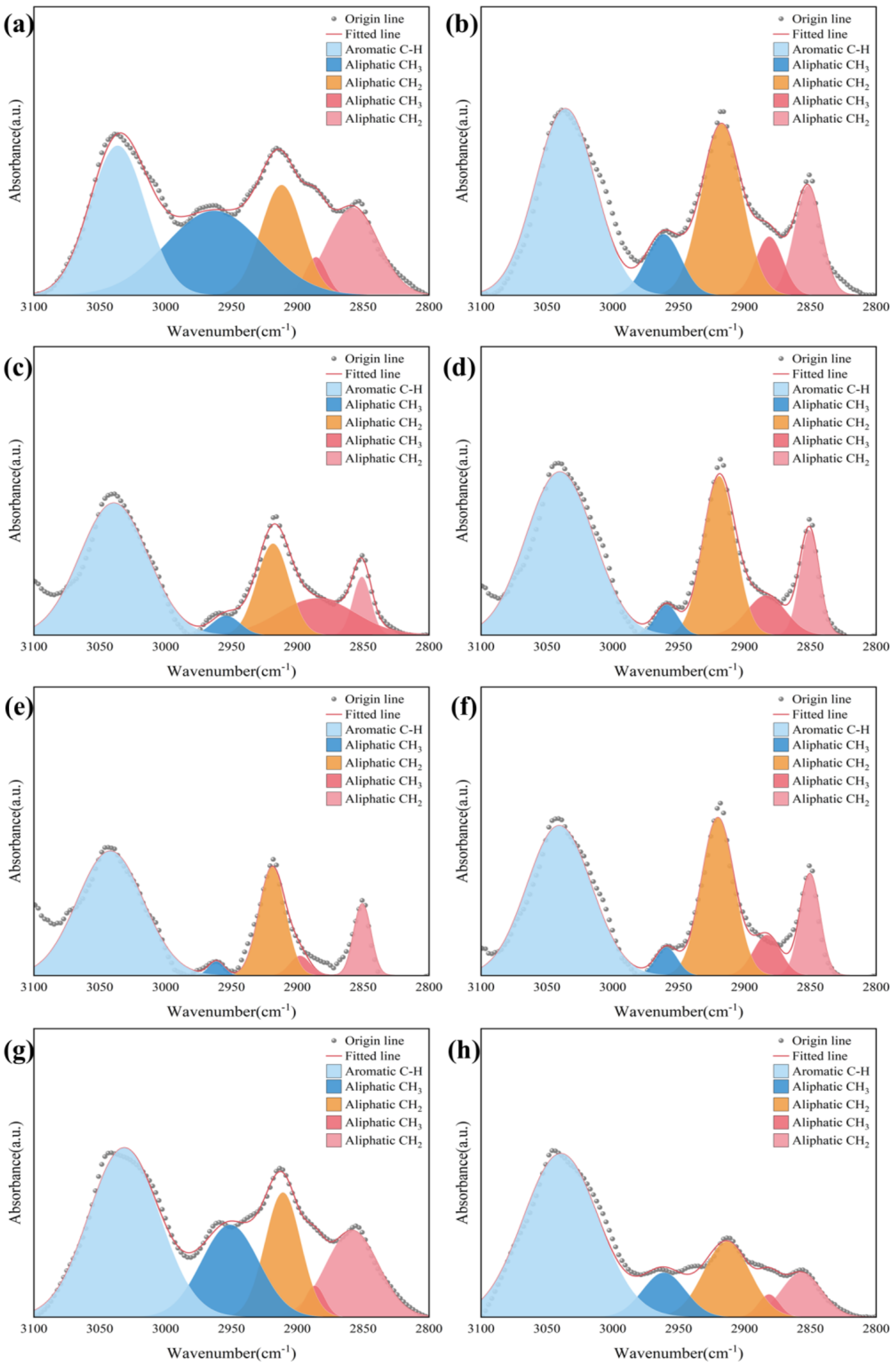

Figure S2. The fitted curve of FTIR of mesophase pitch at 3100-2800 cm^{-1}: (a) MP-1-12, (b) MP-1-14, (c) MP-1-16, (d) MP-2-10, (e) MP-2-12, (f) MP-2-14, (g) MP-3-8, (h) MP-3-10.

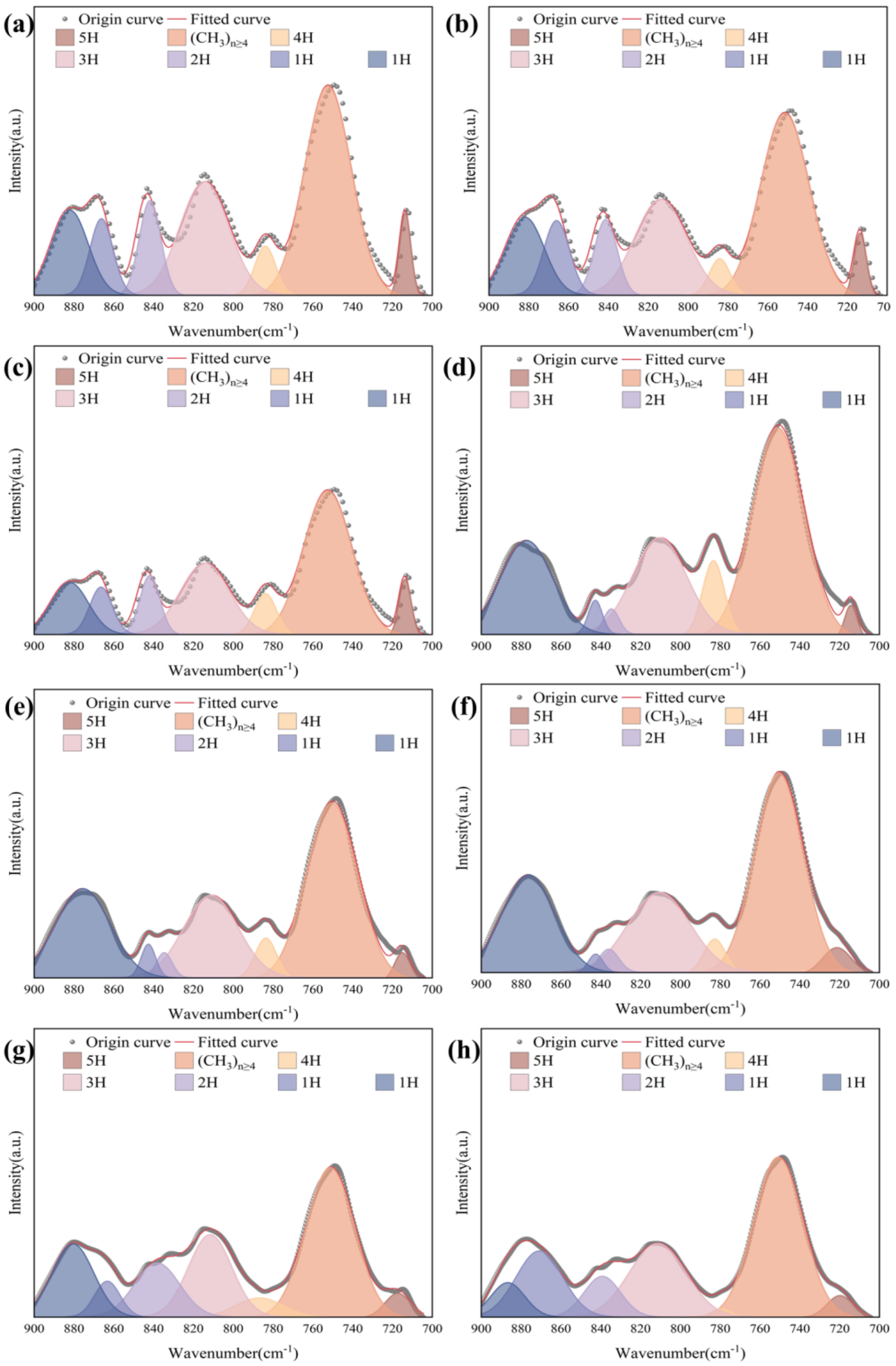

Figure S3. The fitted curve of FTIR of mesophase pitch at 900-700 cm^{-1}: (a) MP-1-12, (b) MP-1-14, (c) MP-1-16, (d) MP-2-10, (e) MP-2-12, (f) MP-2-14, (g) MP-3-8, (h) MP-3-10.

Due to the differences in the molecular weight of the feedstocks, the multimer peaks of each mesophase pitch shift to higher mass ranges and broaden as the fraction becomes heavier. The specific ranges of these peaks are listed in Table S7. The m/z ranges for the multimer peaks of MP-1-16, MP-2-14, and MP-3-12 increase from 230 Da to 265 Da and 280 Da, respectively, which is attributed to the higher molecular weight of FDO-3 compared to FDO-1 and FDO-2.

Table S7. Distribution of polymers peak positions in mesophase pitch

Sample	m/z range						
	**Monomer**	**dimer**	**trimer**	**tetramer**	**pentamer**	**hexamer,**	**heptamer**
MP-1-16	120-350	350-580	580-810	810-1040	1040-1270	1270-1500	1500-1730
MP-2-14	115-380	380-645	645-910	910-1175	1175-1440	1440-1705	1705-1970
MP-3-12	120-400	400-680	680-960	960-1240	1240-1520	1520-1800	1800-2080

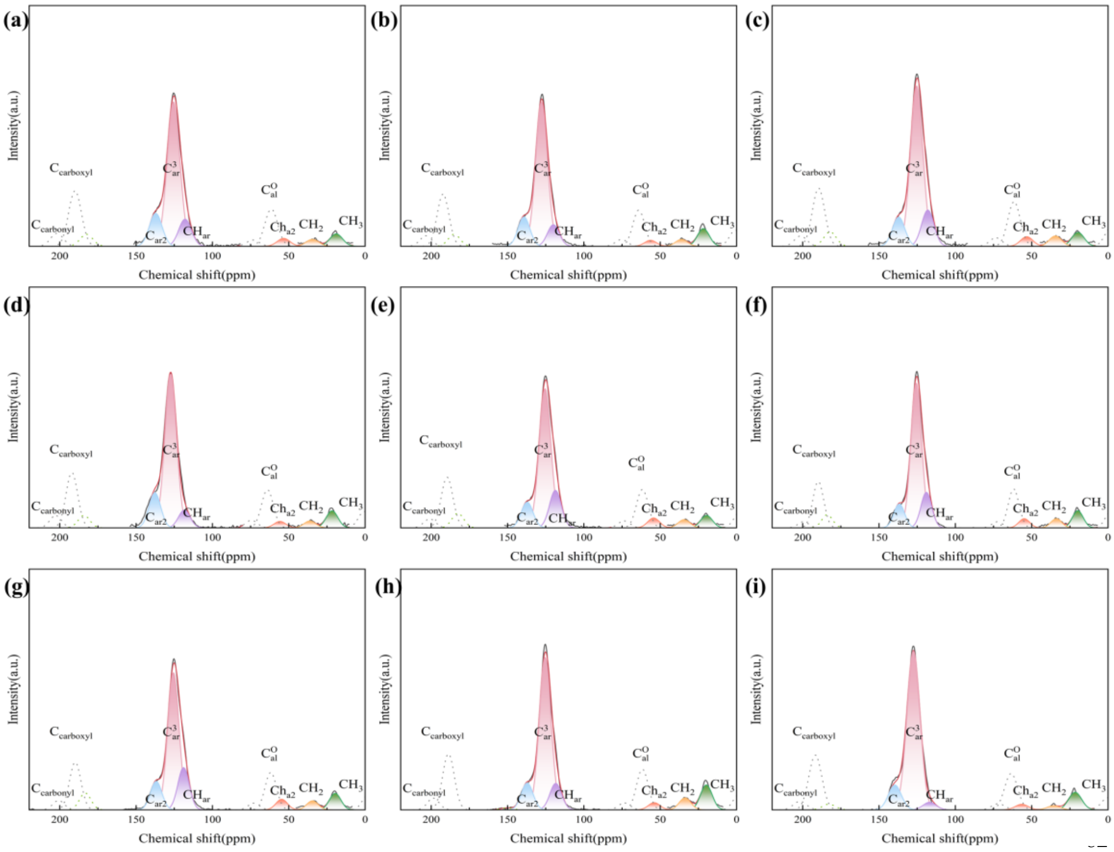

Figure S4. The fitted curve of ^{13}C NMR of mesophase pitch: (a) MP-1-12, (b) MP-1-14, (c) MP-1-16, (d) MP-2-10, (e) MP-2-12, (f) MP-2-14, (g) MP-3-8, (h) MP-3-10, (i) MP-3-12.

$$d_{002} = \frac{\lambda}{2\sin\theta_{002}} \quad \text{(S7)}$$

$$L_c = \frac{0.89\lambda}{\beta_{002}\cos\theta_{002}} \quad \text{(S8)}$$

$$N = \frac{L_c}{d_{002}} + 1 \quad \text{(S9)}$$

In the above equation, λ represents the wavelength of the incident X-ray; θ denotes the Bragg diffraction angle; β is the full width at half maximum (FWHM); d_{002} refers to the interlayer spacing of the aromatic layers; L_c indicates the stacking thickness of the aromatic layers; and N corresponds to the number of stacked aromatic layers.

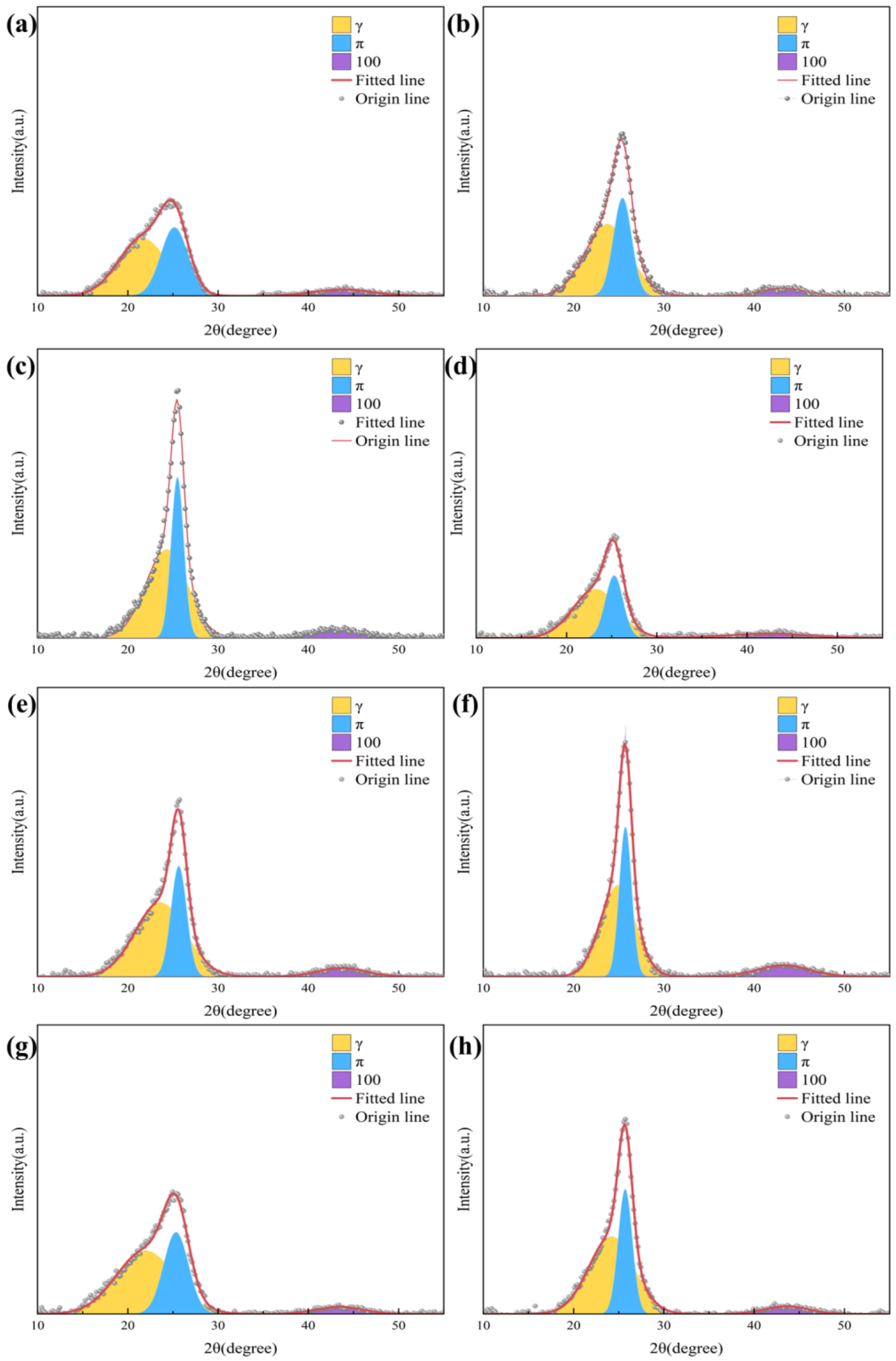

Fig. S5. The fitted curves of XRD spectra of mesophase pitch: (a) MP-1-12; (b) MP-1-14; (c) MP-1-16; (d) MP-2-10; (e) MP-2-12; (f) MP-2-14; (g) MP-3-8; (h) MP-3-10

Table S8. Band assignments of different vibration modes in Raman spectrum

Band	Raman shift(cm^{-1})	vibration mode
G	1580	ideal graphitic lattice (E_{2g}-symmetry)
D1	1350	disordered graphitic lattice (graphene layer edges,A_{1g} symmetry)

D2	1620	disordered graphitic lattice (surface graphene layers, E_{2g}-symmetry)
D3	1500	amorphous carbon (Gaussian line shape)
D4	1200	disordered graphitic lattice (A_{1g} symmetry), polyenes, ionic impurities

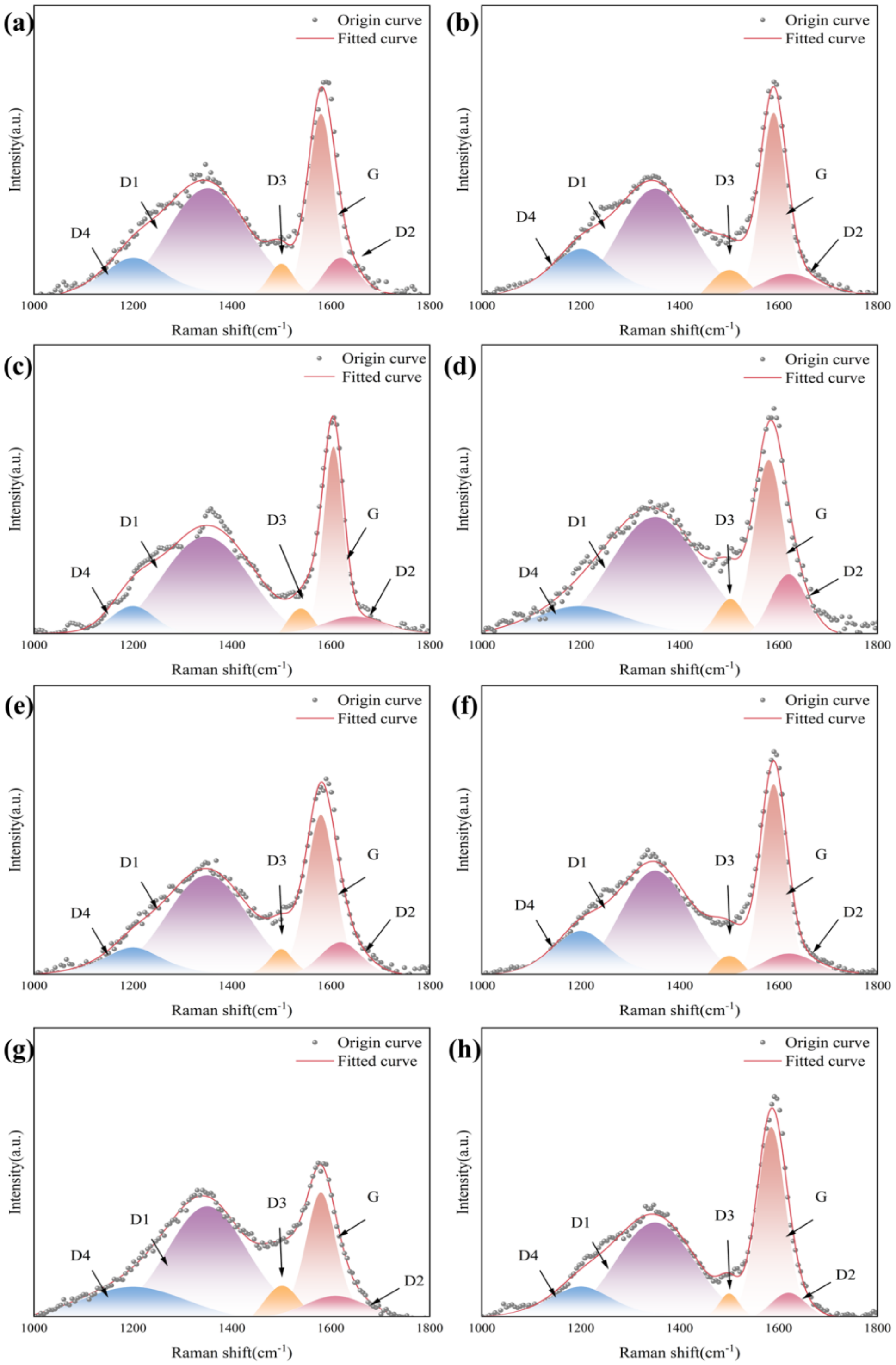

Fig. S6. Raman spectra and fitted curves of mesophase pitch: (a) MP-1-12; (b) MP-1-14; (c) MP-1-16; (d) MP-2-10; (e) MP-2-12; (f) MP-2-14; (g) MP-3-8; (h) MP-3-10

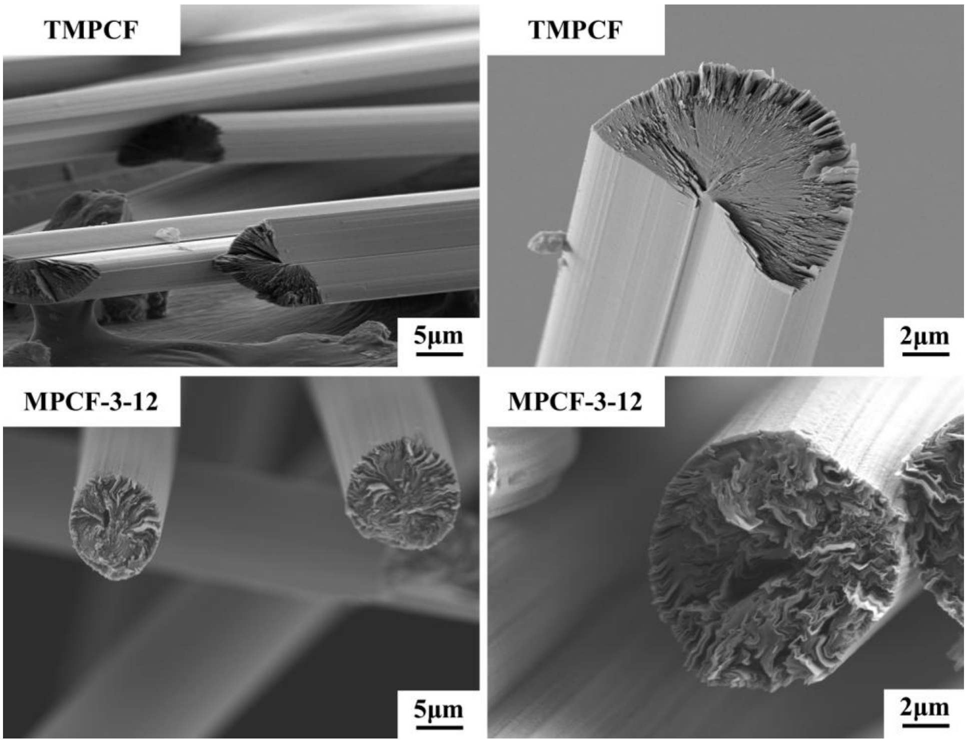

Fig. S7. Typical SEM micrographs of (a) TMPCF; (b) MPCF-3-12.
